# Bispecific antibodies targeting TL1A and IL-12/23p40 suppress pathogenic Th1/Th17 responses and ameliorate colitis in mice

**DOI:** 10.1016/j.isci.2026.117795

**Published:** 2026-10-09

**Authors:** Xia Li, Hao Peng, Feng Hao, Yi Gu, Jinying Ning, Changyuan Yu

**Affiliations:** 1 College of Life Science and Technology, Beijing University of Chemical Technology, Beijing 100029, China; 2 Pharmaceutical Research and Development Department, Trimabs Pharmaceutical Technology (Beijing) Limited, Beijing 101111, China

**Keywords:** bispecific antibodies, common light chain, TL1A, IL-12, IL-23, inflammatory bowel disease

## Abstract

Inflammatory bowel disease (IBD) remains a substantial therapeutic challenge, largely due to the limitations of single-target monotherapies. Here, we engineered two bispecific antibodies, KA-4316 and its half-life-extended derivative KA-5817, which co-target TL1A and IL-12/23p40 utilizing a common light chain design and knobs-into-holes Fc engineering. To improve clinical translatability, KA-5817 was modified with YTE Fc mutations to enhance FcRn binding. KA-4316 exhibited high binding affinity for both targets, high monomer purity, and favorable thermal stability. *In vitro* functional assays demonstrated that KA-4316 potently blocked TL1A-DR3 and IL-12/23p40-IL-12Rβ1 interactions, suppressing downstream signaling and pro-inflammatory cytokine release. *In vivo*, KA-5817 alleviated pathological severity in dextran sulfate sodium (DSS)-induced colitis in TL1A/IL23A/IL12B triple-humanized mice and showed a prolonged serum half-life in FcRn-humanized mice. KA-4316 and KA-5817 exhibit beneficial manufacturability and favorable biological activity. Our results support dual inhibition of TL1A and IL-12/23p40 as a potential therapeutic strategy for IBD.

## Highlights

• KA-4316 efficiently blocks TL1A/IL-12/IL-23-mediated inflammatory responses *in vitro*
• YTE-modified KA-5817 shows extended half-life and favorable preclinical PK profiles
• KA-5817 ameliorates DSS-induced colitis in triple-humanized mice
• Dual TL1A/IL-12/23p40 targeting inhibits pathogenic Th1/Th17 intestinal inflammation

## Graphical abstract

**Figure:**
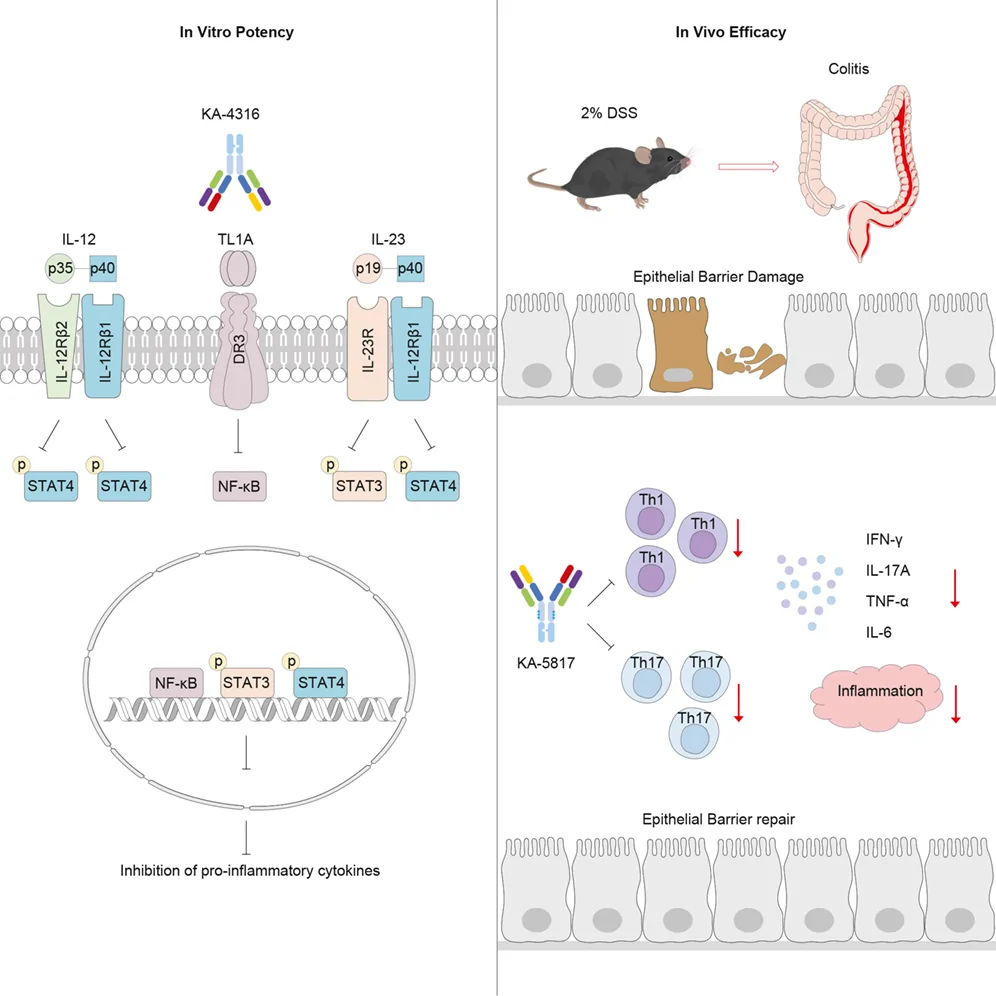

## Teaser

Health sciences; Medicine; Internal medicine; Gastroenterology; Medical manufactured object; Natural sciences; Biological sciences; Immunology; Immunology specialty

## Introduction

Inflammatory bowel disease (IBD) comprises a heterogeneous group of chronic, relapsing intestinal inflammatory disorders driven by dysregulated mucosal immune activation, disrupted epithelial barrier function, and altered host-microbiota interactions.^1^ IBD, including Crohn’s disease (CD) and ulcerative colitis (UC), typically manifests during adolescence or early adulthood and imposes a heavy global health and socioeconomic burden. Epidemiological data indicate that IBD affects approximately 8 million people worldwide, with prevalence continuing to rise in industrialized nations.^2^ Despite remarkable advances in therapeutic development over the past two decades, many patients fail to achieve durable clinical remission or mucosal healing with existing treatments, highlighting a critical unmet need for safer, more effective, and mechanism-driven therapeutic agents.^3^ Current therapeutic strategies for IBD include conventional medications, oral small-molecule drugs, and biological agents. Conventional therapies, such as aminosalicylates, corticosteroids, and non-specific immunosuppressants, often provide incomplete symptom control and carry substantial safety risks. Oral small-molecule drugs, including Janus kinase (JAK) inhibitors and sphingosine-1-phosphate receptor 1 (S1PR) modulators, offer oral convenience but are associated with systemic immunosuppression and cardiovascular concerns. Clinically approved biologic therapies for IBD mainly include anti-TNF-α, anti-integrin, and anti-IL-12/23 antibodies.^4^ Among these, agents targeting the shared p40 subunit of IL-12 and IL-23 (e.g., ustekinumab) or the IL-23-specific p19 subunit (e.g., risankizumab and guselkumab) have transformed clinical outcomes for many patients. However, up to 30%–50% of patients exhibit primary or secondary non-response to single-target biologics, underscoring the inherent limitations of monotherapeutic approaches in addressing the multifactorial and redundant pathogenesis of IBD. The pathogenesis of IBD involves interconnected immune pathways that drive pathogenic Th1 and Th17 polarization, intestinal inflammation, and tissue damage. Among well-validated therapeutic targets, TL1A (TNFSF15) and the IL-12/23p40 subunit play non-redundant and synergistic roles in intestinal inflammation. TL1A, a member of the TNF superfamily, is the only known ligand for its cognate signaling receptor death receptor 3 (DR3 and TNFRSF25). TL1A is highly upregulated in inflamed intestinal tissues of IBD patients and signals through DR3 to activate NF-κB and MAPK signaling, promoting T cell co-stimulation, inflammatory cytokine secretion, and intestinal fibrosis.^5^^,^^6^ TL1A also interacts with the decoy receptor DcR3, which modulates inflammatory homeostasis.^7^ Preclinical and clinical studies have confirmed that blockade of TL1A signaling attenuates intestinal inflammation and fibrosis, supporting TL1A as a tractable therapeutic target.^8^^,^^9^^,^^10^^,^^11^^,^^12^^,^^13^^,^^14^ IL-12 and IL-23 are heterodimeric cytokines that share the p40 subunit and drive Th1 and Th17 differentiation, respectively.^15^ IL-12 promotes IFN-γ production via STAT4 activation, whereas IL-23 stabilizes Th17 cells and drives IL-17 secretion through STAT3 signaling. Both pathways are central to the initiation and perpetuation of chronic intestinal inflammation.^16^ Genetic and pharmacological evidence has firmly established the IL-12/23 axis as a cornerstone of IBD pathogenesis. Notably, TL1A and IL-12/23 pathways exhibit extensive crosstalk and synergistic activity in driving intestinal inflammation.^17^ TL1A enhances IL-12-mediated Th1 polarization and IL-23-driven Th17 differentiation, forming a feedforward inflammatory loop that amplifies mucosal damage. Simultaneous blockade of both pathways may therefore achieve superior therapeutic efficacy compared with single-pathway inhibition and reduce the risk of treatment resistance. However, therapeutic strategies that concurrently and selectively target TL1A and IL-12/23p40 remain poorly explored. In this study, we developed a common light chain (CLC)-based bispecific antibody (BsAb) KA-4316 that co-targets human TL1A and IL-12/23p40, and its half-life-optimized variant KA-5817 harboring the M252Y/S254T/T256E (YTE) Fc mutation. We performed comprehensive *in vitro* characterization, pharmacokinetic analysis in FcRn-humanized mice and cynomolgus monkeys, and *in vivo* efficacy evaluation in a TL1A/IL23A/IL12B triple-humanized mouse model of dextran sulfate sodium (DSS)-induced colitis. Our data show that dual blockade of TL1A and IL-12/23p40 by KA-4316/KA-5817 suppresses pathogenic Th1/Th17 responses and ameliorates acute intestinal inflammation in a mouse model, supporting further translational investigation of this dual-targeting strategy for IBD treatment.

## Results

### Generation and characterization of a TL1A×IL-12/23p40 BsAb with a CLC architecture

To generate a BsAb co-targeting TL1A and IL-12/23p40, we immunized KY-CLC mice (international publication no.: WO2024169925A1) with recombinant human TL1A or IL-12 proteins. Monoclonal antibodies against each antigen were obtained via hybridoma technology and humanization engineering. The variable heavy chain (VH) and kappa light chain (Vκ) sequences were cloned, and Expi-CHO cells were transiently co-transfected with plasmids encoding the anti-TL1A heavy chain, anti-IL-12/23p40 heavy chain, and a shared CLC. Heterodimerization of the two heavy chains was enforced using knobs-into-holes (KiH) Fc engineering, yielding the dual-specific antibody KA-4316 (Figure 1A). KA-4316 was purified by protein A affinity chromatography followed by size-exclusion chromatography-high-performance liquid chromatography (SEC-HPLC), achieving high expression levels of 478.29 mg/L and exceptional monomer purity of 97.35% (Figure 1B). Sodium dodecyl sulfate-polyacrylamide gel electrophoresis (SDS-PAGE) verified that the intact antibody migrated at the expected molecular weight of 144 kDa. Under reducing conditions, the two distinct heavy chain and CLC were clearly resolved (Figure 1C). Thermal stability assessed by differential scanning fluorimetry (DSF) revealed a melting temperature (Tm) of 68.62°C for KA-4316 (Figures 1D and 1E). Together, these biophysical characterizations establish that KA-4316 displays high purity, robust assembly, and favorable thermal stability.

**Figure 1:**
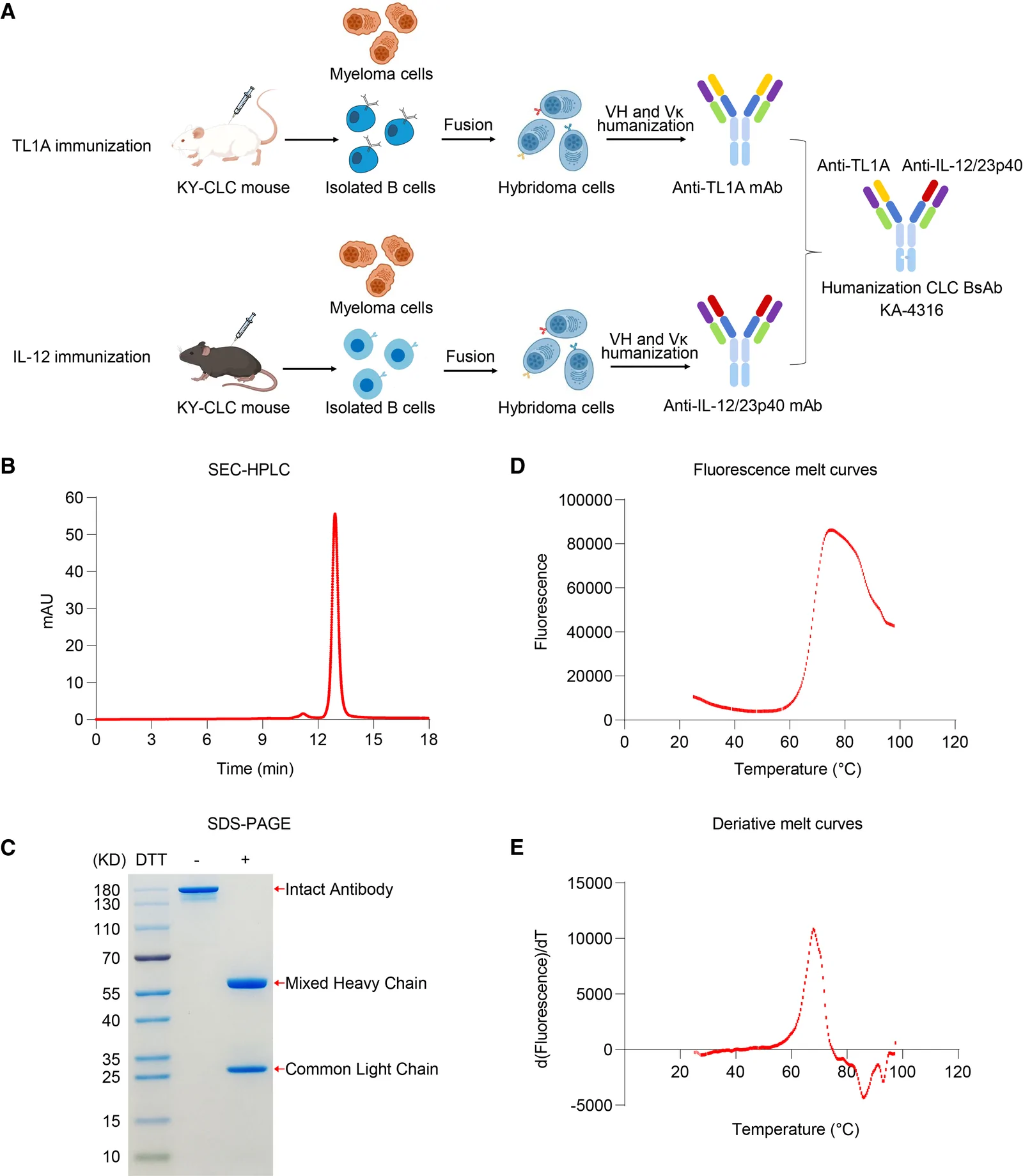
Generation and characterization of the humanized CLC bispecific antibody KA-4316 (A) Schematic overview of the generation of KA-4316. KY-CLC mice were immunized with TL1A or IL-12 antigens. Isolated B cells from immunized mice were fused with myeloma cells to generate hybridoma cells, from which anti-TL1A and anti-IL-12/23p40 monoclonal antibodies (mAbs) were obtained. The variable heavy chain (VH) and variable kappa light chain (Vκ) domains of these mAbs were humanized, and subsequently assembled into the BsAb KA-4316. (B) SEC-HPLC analysis of purified KA-4316, demonstrating a single dominant peak indicative of high monomer purity. (C) SDS-PAGE analysis of KA-4316 under non-reducing (-DTT) and reducing (+DTT) conditions. Under non-reducing conditions, the intact antibody is observed. Under reducing conditions, the mixed heavy chain and common light chain components are resolved. (D) Fluorescence melt curve of KA-4316, showing the change in fluorescence intensity as a function of temperature, reflecting the thermal unfolding profile of the antibody. (E) First derivative of the fluorescence melt curve, revealing the Tm and identifying distinct thermal transition events corresponding to the unfolding of different domains within the bispecific antibody.

### KA-4316 binds human TL1A and IL-12/23p40 with high affinity and cross-species reactivity

Binding kinetics measured by biolayer interferometry (BLI) demonstrated that KA-4316 binds recombinant human TL1A (*K*_D_ = 8.72 × 10^−11^ M) and IL-12/23p40 protein (*K*_D_ = 2.50 × 10^−10^ M) with sub-nanomolar affinity (Figures 2A and 2B). Single-antigen capture ELISA further validated robust binding of KA-4316 to human IL-12 (EC_50_ = 0.33 nM) and IL-23 (EC_50_ = 0.43 nM), two heterodimeric cytokines that share the p40 subunit (Figures 2E and S1C). As a TNF superfamily member, TL1A exists in both soluble and membrane-associated forms *in vivo*. Using ELISA with recombinant TL1A and flow cytometry with TL1A-overexpressing 293T cells, we showed that KA-4316 binds both forms with EC_50_ values of 1.55 and 3.34 nM, respectively (Figures 2F and S1D). Cross-species reactivity profiling revealed strong, dose-dependent binding to cynomolgus monkey TL1A and IL-12/IL-23 but not to their murine orthologs, consistent with its suitability for non-human primate pharmacology and translational development. We next carried out reciprocal sequential BLI assays to verify the simultaneous dual-antigen binding capacity of KA-4316 and exclude mutual binding interference between the two antigen-specific arms. The *K*_D_ values derived from these sequential dual-binding measurements were highly consistent with the single-target affinities described above, indicating uncompromised dual-target binding activity (Figures 2C and 2D). For orthogonal validation, two reciprocal sandwich ELISA setups were implemented. Robust target-specific binding signals were detected for KA-4316 in both assays (Figures S1A and S1B). Collectively, these results confirm that KA-4316 can efficiently and simultaneously engage TL1A and IL-12/23p40 without steric hindrance or binding interference. Neutralization ELISA demonstrated that KA-4316 effectively blocks the interaction of both IL-12 and IL-23 with IL-12Rβ1, with IC_50_ values of 12.88 and 16.81 nM, respectively (Figure 2G), confirming potent inhibition of both cytokine signaling axes. TL1A signals through the pro-inflammatory death receptor DR3, which is critical for multiple T cell-driven inflammatory diseases,^12^ and is also sequestered by the decoy receptor DcR3, which modulates other TNF superfamily ligands including FasL and LIGHT.^7^ Competition ELISA revealed that KA-4316 neutralizes TL1A binding to DR3 with an IC_50_ of 2.76 nM and to DcR3 with an IC_50_ of 17.28 nM (Figure 2H), demonstrating that KA-4316 blocks TL1A binding to both DR3 and DcR3, with more potent neutralizing activity against DR3.

**Figure 2:**
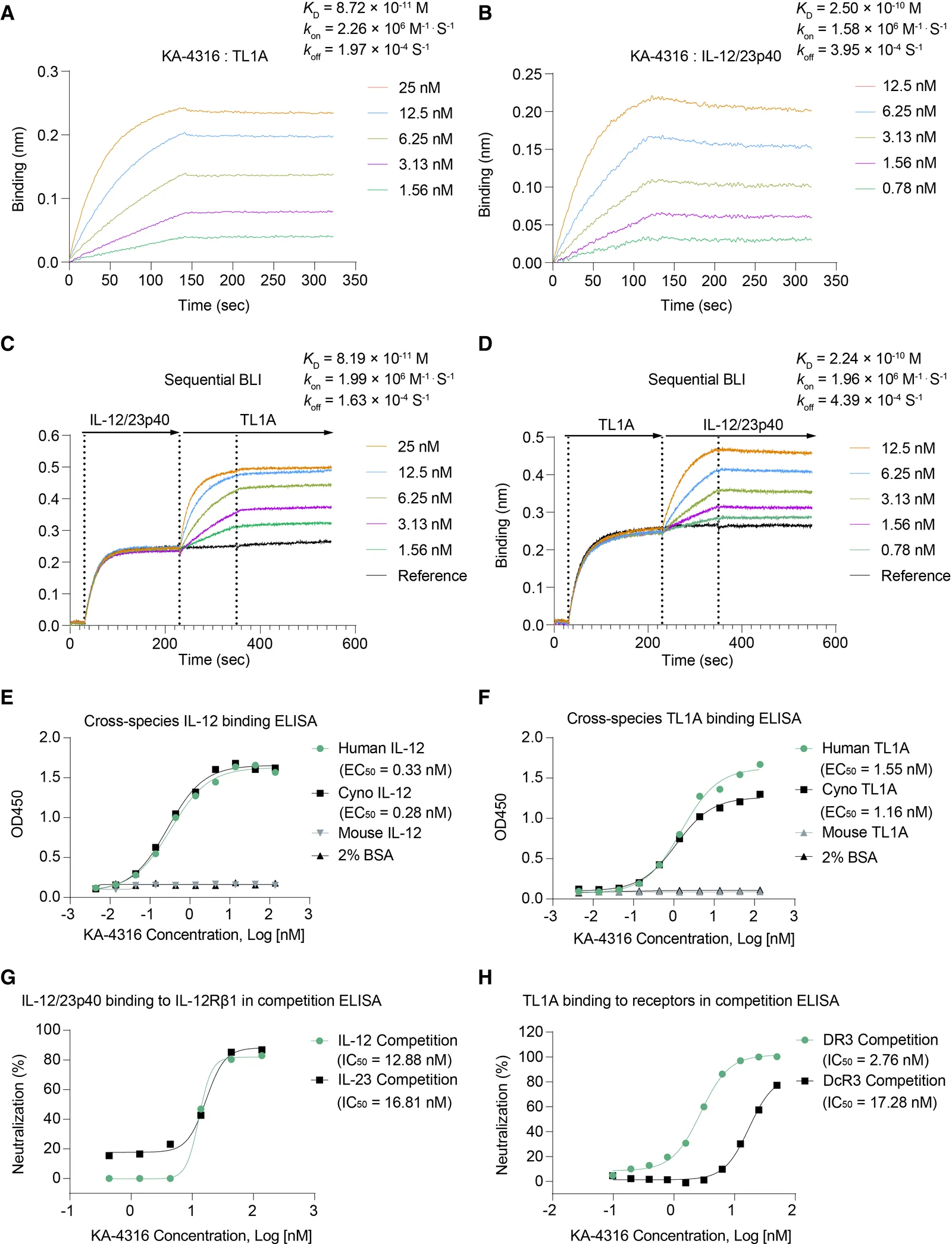
Potent and cross-species binding and functional activity of KA-4316 (A and B) Real-time binding kinetic analysis of KA-4316 antibody interacting with TL1A trimer antigen and IL-12/23p40 antigen on the Octet platform. The association and dissociation traces were fit to a 1:1 binding model. The derived kinetic parameters (*K*_D_, k_on_, and k_off_) indicate high-affinity binding. (C and D) Reciprocal sequential BLI to test concurrent dual-antigen binding. In (C), immobilized KA-4316 was saturated with IL-12/23p40 before measuring secondary TL1A binding. In the reverse assay (D), KA-4316 was pre-saturated with TL1A, followed by detection of IL-12/23p40 binding across serial antigen dilutions. Kinetic constants (*K*_D_, k_on_, and k_off_) derived from each sequential measurement are labeled above each image. (E and F) Cross-species binding characterization of KA-4316. ELISA showing potent binding to human and cynomolgus monkey IL-12 (E) and TL1A (F) with low picomolar EC_50_ values, demonstrating robust cross-species reactivity. Meanwhile, no detectable binding was observed against mouse IL-12 or TL1A. (G and H) Functional competition ELISA assays. (G) KA-4316 efficiently blocks binding of IL-12/23p40 to IL-12Rβ1. (H) KA-4316 blocks TL1A binding to its receptors DR3 or DcR3, with distinct IC_50_ values. Dose-response curves were fitted by nonlinear regression using a four-parameter logistic model. EC_50_ and IC_50_ values are reported with curve-fitted 95% confidence intervals.

### KA-4316 acts as a potent dual inhibitor of TL1A- and IL-12/IL-23-mediated inflammatory responses *in vitro*

DR3, a death domain-containing receptor of the TNFR superfamily primarily expressed on lymphocytes, mediates both NF-κB activation and apoptotic signaling.^18^ In TF1-NF-κB-luciferase-DR3 reporter cells, both KA-4316 (monovalent for TL1A) and the parental anti-TL1A mAb (bivalent) inhibited TL1A-driven signaling in a dose-dependent manner, with IC_50_ values of 0.49 and 0.19 nM, respectively (Figure 3A). In TF-1 cells (human erythroleukemic cell) co-treated with cycloheximide, TL1A elicits DR3-dependent apoptosis.^19^ KA-4316 and parental anti-TL1A antibody neutralized this response with IC_50_ values of 1.82 and 0.68 nM, consistent with monovalent versus bivalent avidity differences (Figure 3B). IL-12 engagement of its heterodimeric IL-12Rβ1/β2 receptor activates JAK2/TYK2 signaling and downstream STAT4,^20^ driving IFN-γ production by NK cells and effector T cells.^21^ In HEK-Blue IL-12 reporter cells, KA-4316 (monovalent for IL-12/23p40) and parental anti-IL-12/23p40 mAb (bivalent) blocked IL-12-induced STAT4 activation with IC_50_ values of 2.79 and 0.34 nM (Figure 3C). Similarly, in NK92MI cells, KA-4316 inhibited IL-12-stimulated IFN-γ secretion with an IC_50_ of 1.95 nM, compared with 0.30 nM for the bivalent parental antibody (Figure 3D). IL-23, another IL-12 family cytokine comprising p40 and p19 subunits, signals via IL-12Rβ1/IL-23R to activate JAK2/TYK2 and STAT3, a pathway central to Th17-driven autoimmunity.^22^ In 293T-STAT3-luciferase reporter cells, stably expressing IL-12Rβ1 and IL-23R, KA-4316 and anti-IL-12/23p40 mAb attenuated IL-23-mediated STAT3 activation with IC_50_ values of 0.29 and 0.07 nM, respectively (Figure 3E). Notably, TL1A has been shown to synergize with IL-12 and IL-18 to amplify IFN-γ production in human T and NK cells.^17^ In cytokine-stimulated human PBMCs, KA-4316 potently suppressed IFN-γ release with an IC_50_ of 0.47 nM, superior to both parental antibodies alone and comparable to their combined administration (IC_50_ = 0.98 nM) (Figure 3F). Collectively, in single-pathway functional assays, only the pathway-matched parental antibody produced robust inhibition, while the non-targeted parental antibody had negligible functional effects. Consistent with this specific monospecific activity, the equimolar mixture (0.5:0.5) displayed an approximately 2-fold higher IC_50_ than the individual parental antibody, reflecting dilution of the active antibody component. In contrast, under dual-pathway co-stimulation conditions, the parental antibody mixture achieved superior inhibitory potency comparable to KA-4316. This rigorous head-to-head functional benchmarking highlights the dual-blockade capability of KA-4316. We further quantified dual-target inhibition relative to single-agent treatment using the Chou-Talalay method via CompuSyn software.^23^ Across multiple effect levels (fraction affected, Fa = 0.5, 0.75, and 0.9), the calculated combination index (CI) values for the parental antibody mixture were below 1, quantitatively verifying pharmacological synergy (Figure S2A). Corresponding isobologram analysis provided orthogonal visual confirmation. All data points lie below the theoretical additive reference line, confirming synergistic functional interaction between the two target pathways (Figure S2B). However, direct head-to-head comparison showed that KA-4316 exhibited only a modest improvement in IC_50_ relative to the combination of the two parental antibodies, and this difference did not reach statistical significance. These observations indicate that although target-level synergy is achieved upon simultaneous blockade of TL1A and IL-12/23p40, no additional functional benefit conferred by the unique bispecific molecular architecture enables KA-4316 to outperform combined monospecific antibody treatment.

**Figure 3:**
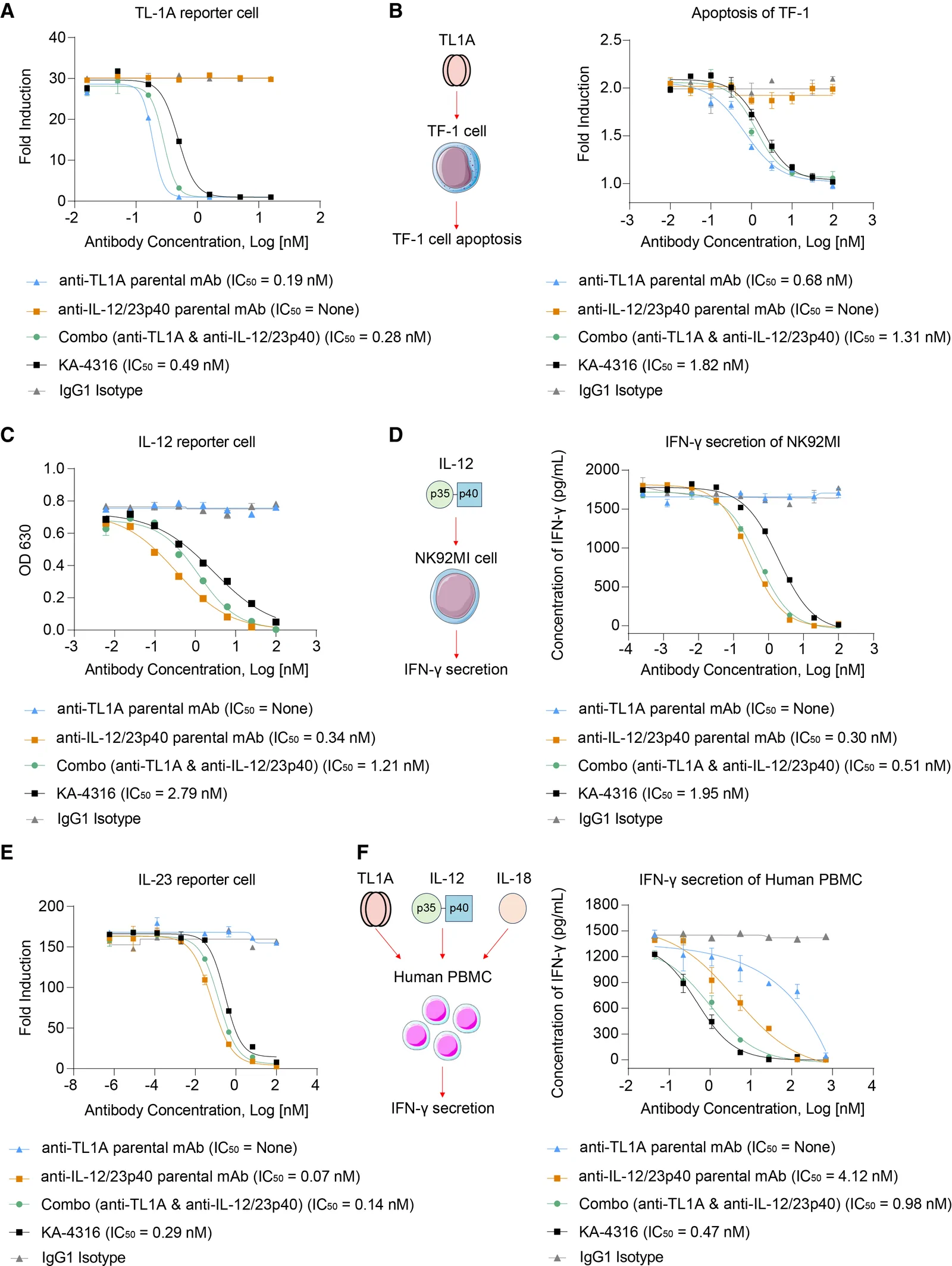
KA-4316 is a potent dual inhibitor of TL1A- and IL-12/23-mediated inflammatory signaling *in vitro* (A) Inhibition of TL1A-induced NF-κB activation in TF-1-NF-κB-Luciferase-DR3 reporter cells. Cells were treated with serially diluted antibodies, and luciferase activity was measured. (B) Neutralization of TL1A-induced apoptosis in cycloheximide-treated TF-1 cells. Apoptosis was assessed by luminescence-based assay. (C) Inhibition of IL-12-induced STAT4 activation in HEK-Blue IL-12 reporter cells. Activity was measured by SEAP reporter assay (optical density 630 (OD630) nm). (D) Neutralization of IL-12-induced IFN-γ secretion in NK92MI cells. IFN-γ levels in culture supernatants were quantified by ELISA. (E) Inhibition of IL-23-induced STAT3 activation in 293T-STAT3-Luciferase reporter cells expressing IL-12Rβ1/IL-23R. Luciferase activity was measured. (F) Neutralization of synergistic IFN-γ production induced by TL1A, IL-12, and IL-18 in human PBMCs. IFN-γ secretion was measured by ELISA. All images include four test groups: KA-4316 bispecific, anti-TL1A parental mAb alone, anti-IL-12/23p40 parental mAb alone, and their equimolar parental mixture (combo). IgG1 isotype served as negative control in all assays. Dose-response curves were fitted by nonlinear regression using a four-parameter logistic model. IC_50_ values are reported with curve-fitted 95% confidence intervals.

### KA-5817 harboring the YTE mutation exhibits prolonged half-life in FcRn-humanized mice

The neonatal Fc receptor (FcRn) governs IgG recycling and antibody half-life extension.^24^ FcRn-humanized mice, which replace the murine *Fcgrt* gene with human *FCGRT*, facilitate reliable preclinical profiling of therapeutic antibody pharmacokinetics. The triple Fc mutation YTE enhances binding to human and cynomolgus FcRn at pH 6.0 (without impairing antigen binding or function) and extends serum half-life nearly 4-fold in non-human primates.^25^ To improve Pharmacokinetics (PK) properties of KA-4316, we introduced the YTE mutations into its heavy chain to generate the BsAb KA-5817. KA-5817 was purified by protein A affinity chromatography followed by SEC-HPLC, achieving an expression yield of 282.46 mg/L in ExpiCHO cells and a purity of 95.67% (Figure 4B). SDS-PAGE confirmed that the intact antibody migrated at the expected molecular weight of 144 kDa (Figure 4A). We assessed PK profiles of KA-4316 and KA-5817 in homozygous FcRn-humanized mice. Mice received a single intravenous injection of 10 mg/kg antibody and serum concentrations were measured by ELISA at designated time points (Figure 4C). KA-5817 exhibited a serum half-life of 21.53 ± 5.84 days, compared with 8.32 ± 0.80 days for KA-4316 (Figure 4D), verifying that the YTE mutation substantially prolongs BsAb persistence in FcRn-humanized mice. Additional non-compartmental PK parameters (maximum concentration [Cmax] and area under the curve [AUC_0-∞_]) are listed in Figure 4E, reflecting elevated systemic exposure for KA-5817.

**Figure 4:**
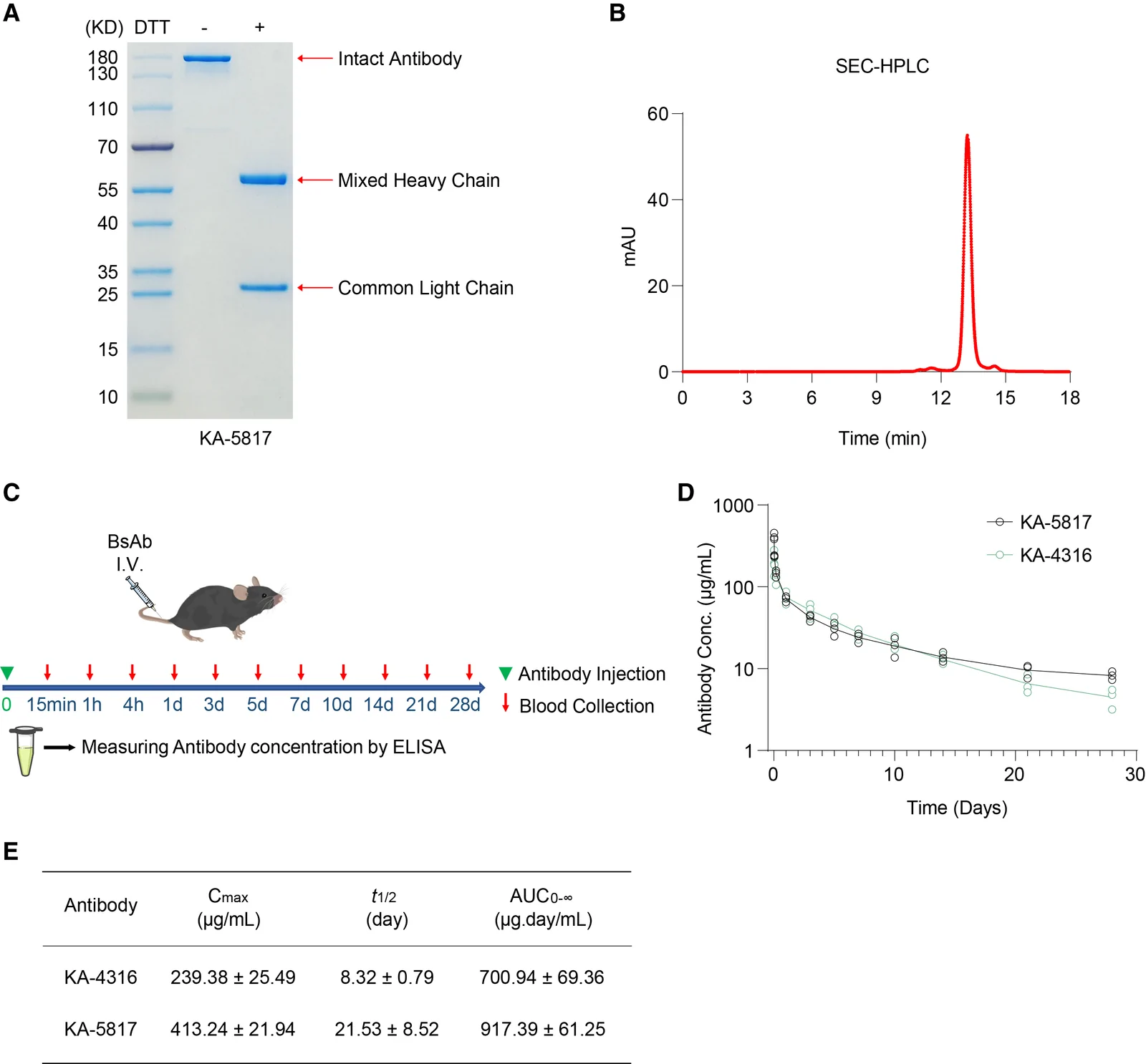
Biophysical characterization and pharmacokinetic evaluation of KA-5817 in FcRn-humanized mice (A) SDS-PAGE analysis of purified KA-5817 under non-reducing and reducing conditions. The intact antibody (144 kDa) is observed under non-reducing conditions, while the mixed heavy chains (60 kDa) and common light chain (25 kDa) are resolved under reducing conditions. (B) SEC-HPLC profile of KA-5817, showing a single major peak corresponding to the monomeric antibody species, confirming high purity. (C) Schematic of the pharmacokinetic study design in FcRn-humanized mice. KA-5817 or KA-4316 was administered as a single intravenous (i.v.) injection at 10 mg/kg (*n* = 3 animals per group), with blood samples collected at predefined time points (0, 15 min, 1 h, 4 h, 1 day, 3 days, 5 days, 7 days, 10 days, 14 days, 21 days, and 28 days). Serum antibody concentrations were quantified by ELISA. (D) Serum concentration-time profiles of KA-5817 and KA-4316 following a single intravenous dose (10 mg/kg) in FcRn-humanized mice. KA-5817 exhibits a significantly prolonged half-life compared to KA-4316. (E) Summary table of key non-compartmental pharmacokinetic parameters, including maximum observed concentration (Cmax), terminal elimination half-life (*t*_1/2_), and average extrapolated area under the concentration-time curve from time zero to infinity (AUC_0-∞_). AUC unit: μg·day/mL. All values are presented as mean ± SEM.

### Pharmacokinetic and toxicological profiles of KA-5817 in cynomolgus monkeys

FcRn mediates salvage and recycling of IgG, thereby governing antibody serum half-life. Owing to high conservation of FcRn function and binding specificity between cynomolgus monkeys and humans, non-human primate PK studies enable robust prediction of human antibody disposition. However, therapeutic human antibodies may elicit immunogenicity in monkeys due to their xenogeneic nature. Here we performed a non-good laboratory practice (GLP) preclinical study to assess the acute toxicity and PK properties of KA-5817 following a single intravenous administration in cynomolgus monkeys. Animals received doses of 3 or 10 mg/kg, and serum concentrations of intact BsAb were quantified by ELISA at defined time intervals (Figure 5A). Serum concentration-time profiles of KA-5817 were evaluated (Figure 5B). Pharmacokinetic parameters were derived by non-compartmental analysis performed in Phoenix WinNonlin v.8.3. The terminal elimination half-life was calculated from the log-linear decay phase fitted using three sequential sampling time points following Cmax (day 5, day 7, and day 14). Day 21 measurements were excluded due to anti-drug antibody (ADA) interference that distorts elimination curve fitting (Figures S3A and S3B). The full panel of non-compartmental PK outputs, including Cmax, *t*_1/2_, AUC_0-∞_, and systemic clearance (CL), is summarized in Figure 5C. PK analysis revealed dose-dependent divergence in elimination kinetics. While the two doses produced comparable overall AUC_0-∞_, the 10 mg/kg group exhibited a higher peak concentration, accelerated systemic clearance, and a shorter terminal half-life relative to the 3 mg/kg group. We therefore monitored ADA formation against KA-5817 across a 21-day sampling period in cynomolgus monkeys using a KA-5817 capture ELISA. The 10 mg/kg dose elicited earlier and more robust ADA signals than the 3 mg/kg group. This augmented ADA response at high dosage coincides with enhanced drug clearance and shortened terminal half-life, establishing that ADA formation drives the observed dose-dependent alterations in KA-5817 pharmacokinetics (Figures S3C and S3D). Comprehensive evaluation of hematological parameters and serum biochemistry revealed no evidence of treatment-related hematologic, hepatic, or renal toxicity at either dose level. Together, these findings define the PK and acute safety profile of KA-5817 in cynomolgus monkeys and support its continued clinical development.

**Figure 5:**
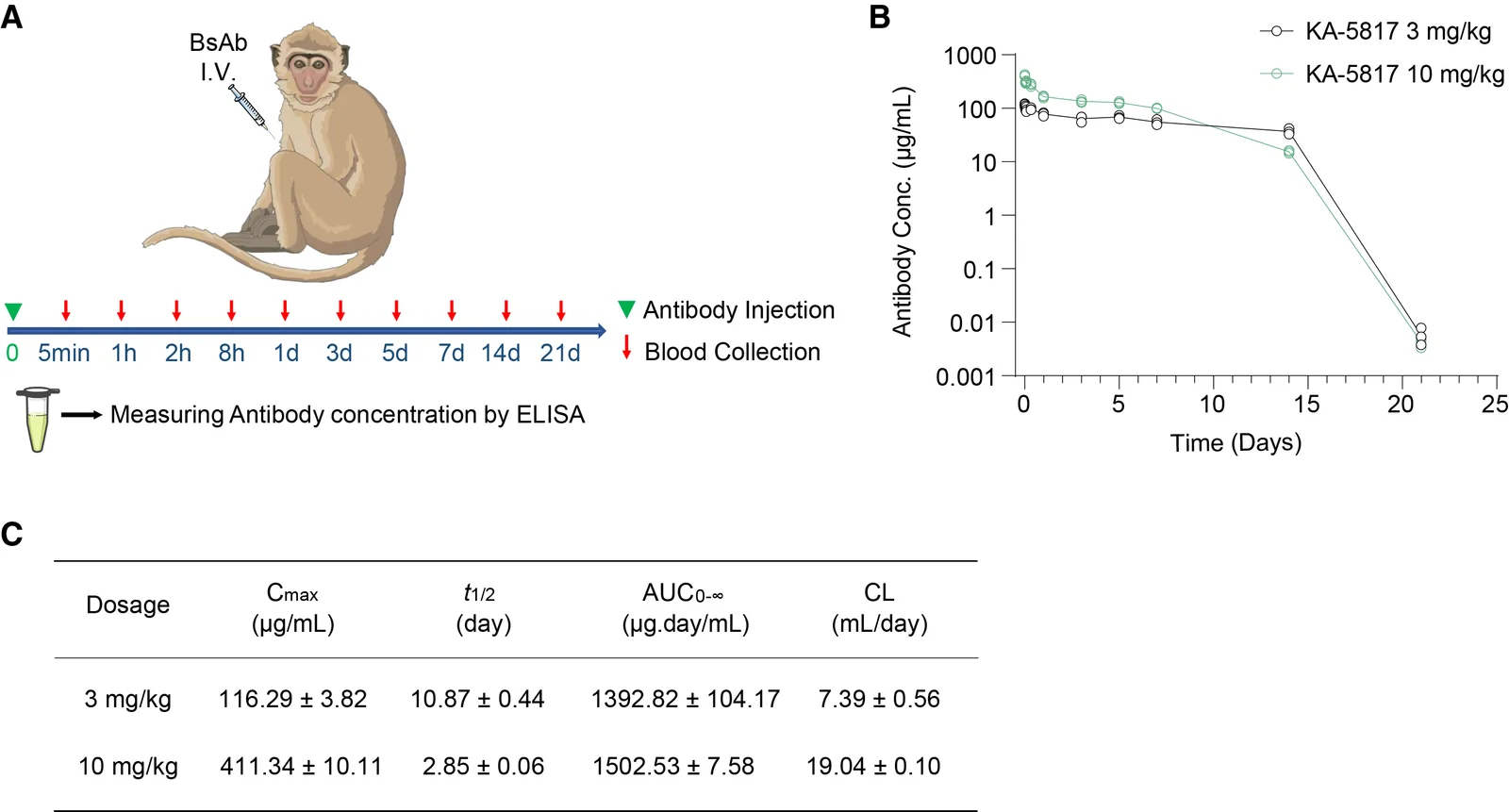
Pharmacokinetic profile of KA-5817 in cynomolgus monkeys (A) Schematic overview of the pharmacokinetic study design in cynomolgus monkeys. Animals received a single intravenous (i.v.) injection of KA-5817 at either 3 mg/kg or 10 mg/kg (*n* = 1 animal per group). Blood samples were collected at predefined time points: 5 min, 1 h, 2 h, 8 h, 1 day, 3 days, 5 days, 7 days, 14 days, and 21 days post-administration. Serum concentrations of intact KA-5817 were quantified by ELISA. (B) Serum concentration-time profiles of KA-5817 in cynomolgus monkeys following i.v. dosing at 3 and 10 mg/kg. (C) Summary table of key non-compartmental pharmacokinetic parameters, including Cmax, *t*_1/2_, AUC_0-∞_, and CL. AUC unit: μg·day/mL; clearance unit: mL/day. Pilot cynomolgus monkey PK study to characterize systemic exposure trends, with one animal per group and no biological replicates. Plotted values are ELISA technical replicates reflecting only assay variation, presented as mean ± SEM. No statistical analysis was performed.

### KA-5817 potently ameliorates pathological severity in a TL1A/IL23A/IL12B triple-humanized mouse model of DSS-induced colitis

We evaluated the *in vivo* therapeutic efficacy of KA-5817 in a clinically translatable DSS-induced acute colitis model using TL1A/IL23A/IL12B triple-humanized C57BL/6 mice (7–9 weeks old, male) housed under specific-pathogen-free (SPF) conditions. Acute colitis was induced by 2% (w/v) DSS in drinking water for 6 days, followed by 3 days of regular water. Mice were randomized into six groups (*n* = 6 per group): naive triple-humanized controls (no DSS), DSS-challenged mice treated with IgG1 isotype control, monospecific anti-TL1A mAb, monospecific anti-IL-12/23p40 mAb, a combination of anti-TL1A plus anti-IL-12/23p40 mAbs (0.5: 0.5 Combo), and bispecific KA-5817 (total 20 mg/kg, intravenously on days 0, 3, and 7). Clinical disease activity index (DAI), comprising body weight loss, stool consistency, and rectal bleeding (scored 0–4 per metric, total 0–12), was monitored daily. Mice were sacrificed on day 9 for colon length analysis and histological assessment (Figure 6A). Relative to naive animals, all DSS-exposed antibody control groups exhibited severe colitis characterized by progressive weight loss, persistently elevated DAI scores, and pronounced colon shortening consistent with severe colitis pathology. Treatment with individual monospecific anti-TL1A or anti-IL-12/23p40 antibodies yielded only partial alleviation of intestinal injury. Strikingly, KA-5817 achieved robust disease suppression comparable to the equimolar physical mixture of the two monospecific antibodies (Figures 6B–6E). Histological H&E staining revealed extensive epithelial erosion, crypt loss, and marked inflammatory cell infiltration within the colonic mucosa of isotype-treated mice compared with naive mice. While single monospecific antibodies offered limited mucosal protection, KA-5817 and the combo group effectively preserved intestinal architecture, maintaining intact crypt structure and substantially reducing immune cell infiltration (Figure 6F). Collectively, these head-to-head *in vivo* comparisons demonstrate that the BsAb KA-5817 exerts significantly enhanced therapeutic efficacy over single-target monospecific antibodies. These findings indicate that the robust anti-inflammatory activity of KA-5817 relies on simultaneous dual blockade of the TL1A and IL-12/23 pathways, supporting further translational evaluation of dual-pathway inhibition as a strategy for the treatment of IBD.

**Figure 6:**
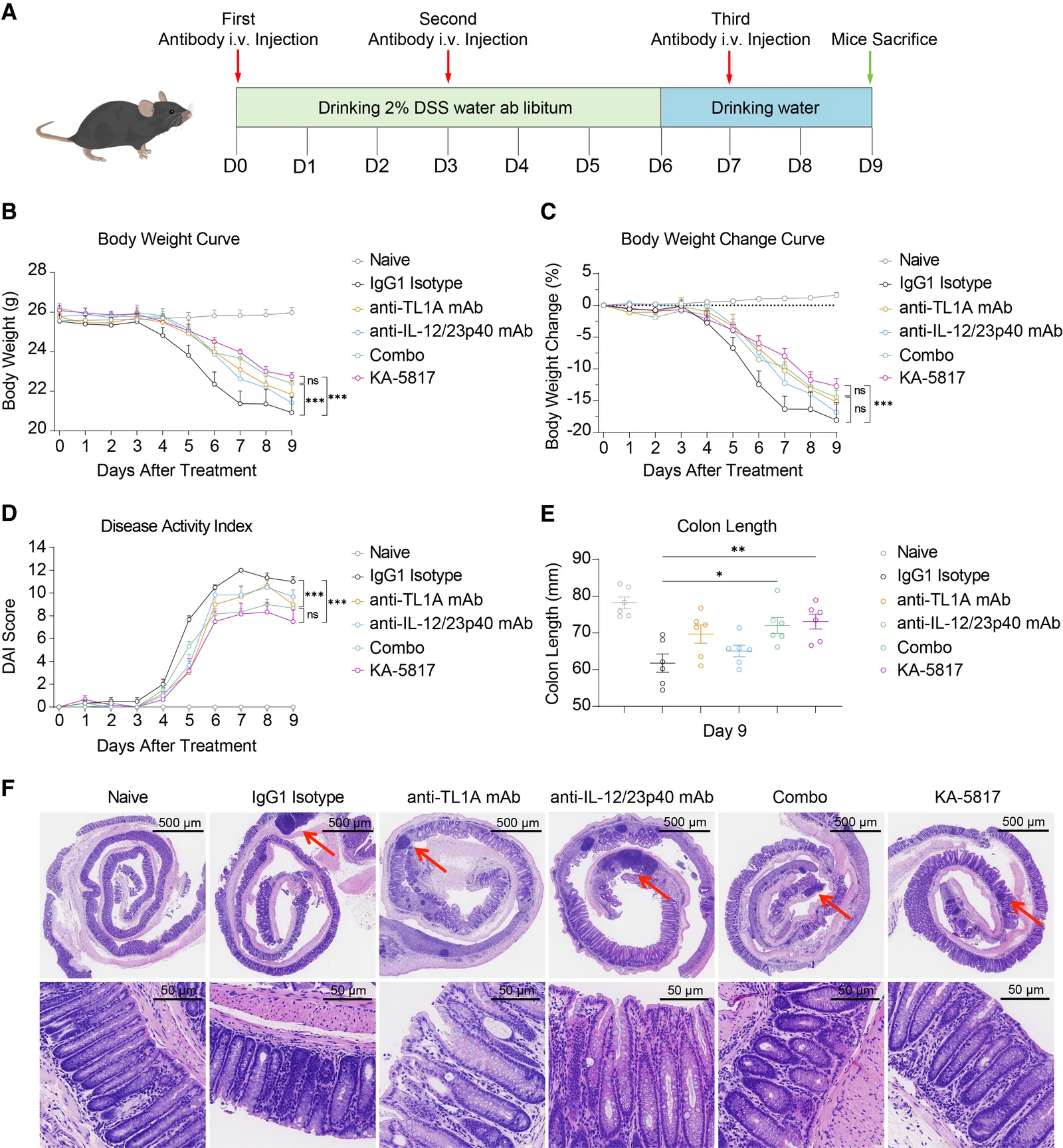
KA-5817 ameliorates disease pathology in a TL1A/IL23A/IL12B triple-humanized mouse model of DSS-induced colitis (A) Schematic overview of the acute colitis model and antibody treatment regimen. Triple-humanized mice received 2% DSS in drinking water from day 0 to day 6, followed by regular water. Equimolar antibodies were administered intravenously on days 1, 3, and 7. Mice were sacrificed on day 9 for analysis. (B and C) Body weight changes over the course of the study. Absolute body weight curves (B) and percentage of body weight change curves (C) show that antibody-treated mice maintain significantly higher weight compared with the IgG1 isotype group, with the naive group showing no weight loss. (D) Daily DAI scores reflecting comprehensive intestinal disease severity. (E) Quantitative analysis of colon length at the experimental endpoint. (F) Representative H&E staining and histological evaluation of colonic mucosal tissues from each group, showing epithelial integrity, crypt structure, and inflammatory infiltration (red arrows). Scale bars, 500 μm (low magnification) and 50 μm (high magnification). Six experimental groups were included (*n* = 6 animals per group): naive control, DSS-induced colitis with IgG1 isotype control, single anti-TL1A mAb monotherapy, single anti-IL-12/23p40 mAb monotherapy, equimolar combination of anti-TL1A and anti-IL-12/23p40 mAbs, and bispecific KA-5817 treatment. Data are presented as mean ± SEM. Body weight, relative body weight, and DAI scores were analyzed using two-way repeated-measures ANOVA. Colon length was compared using one-way ANOVA. ∗*p* ≤ 0.05, ∗∗*p* ≤ 0.01, and ∗∗∗*p* ≤ 0.001. ns, no significance.

### KA-5817 suppresses pathogenic Th1/Th17 responses and mitigates pro-inflammatory signaling in DSS-induced colitis

To dissect the cellular and molecular mechanisms underlying KA-5817-mediated intestinal protection, we analyzed immune cell populations and inflammatory mediators within the colonic lamina propria of DSS-challenged triple-humanized mice. Flow cytometry was used to quantify CD4^+^ T lymphocyte subsets driving intestinal inflammation (Figure 7A). Compared with the IgG1 isotype group, monotherapy with anti-TL1A or anti-IL-12/23p40 mAb induced partial reductions in pathogenic Th1 (CD4^+^ IFN-γ^+^) and Th17 (CD4^+^ IL-17A^+^) cell frequencies. KA-5817 treatment significantly reduced the frequency of Th1 cells, while Th17 subsets showed a trend toward reduction (Figures 7B and 7C). No statistically significant intergroup differences were observed for the proportion of regulatory T cells (CD4^+^ FOXP3^+^) (Figure 7D). We next quantified pro-inflammatory cytokines and intracellular signaling markers from colon tissue homogenates. Elevated tissue TNF-α and IL-6 concentrations observed in isotype-treated colitic mice were substantially reduced by KA-5817 (Figures 7E and 7F). KA-5817 also diminished STAT3 phosphorylation levels (Figure 7G). Collectively, these mechanistic data demonstrate that dual blockade of TL1A and IL-12/23 by KA-5817 restrains pathogenic Th1/Th17 responses, lowers local pro-inflammatory cytokine production, and inhibits STAT3 signaling, thereby alleviating intestinal inflammatory injury.

**Figure 7:**
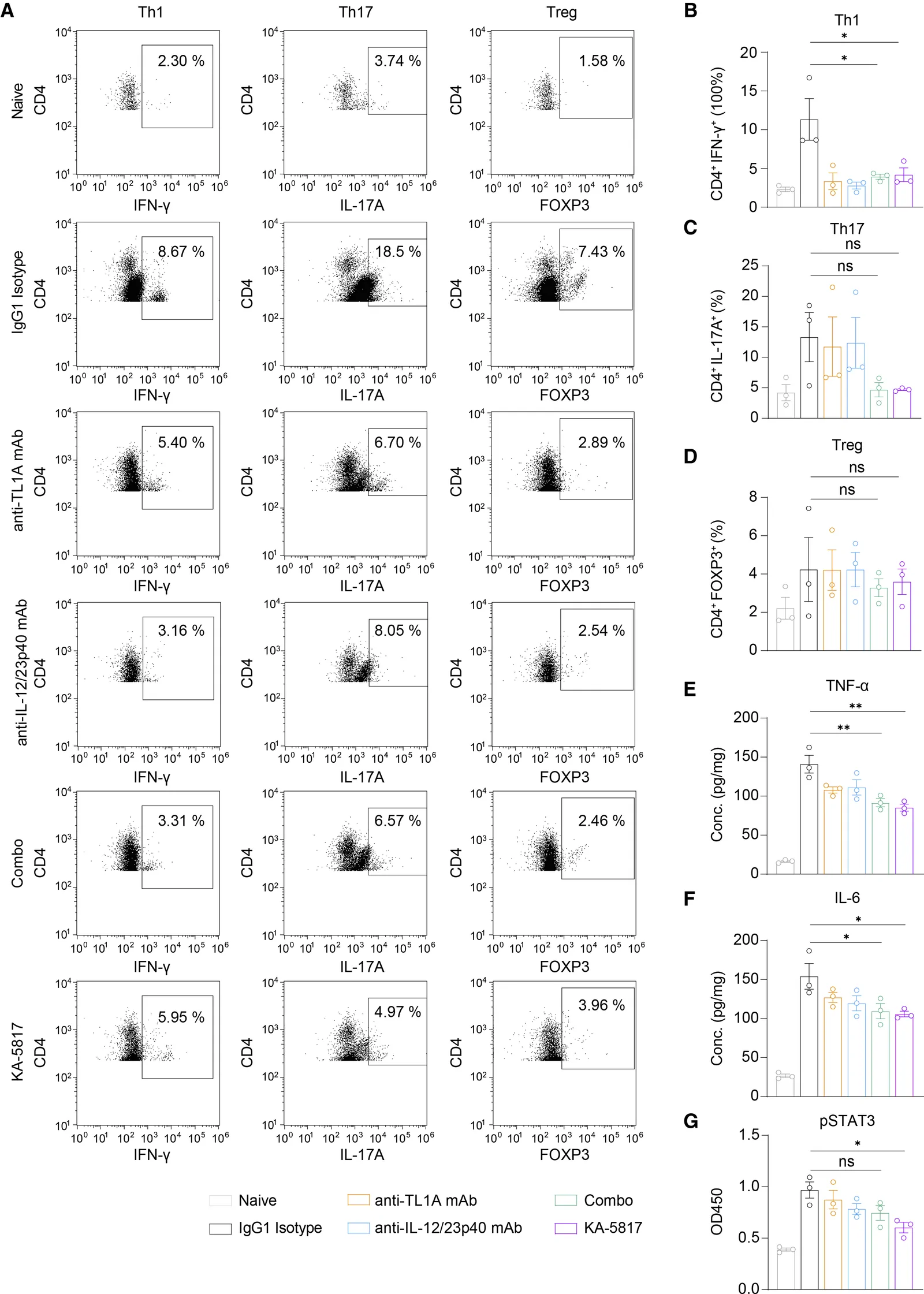
KA-5817 remodels intestinal T cell homeostasis and suppresses pro-inflammatory signaling in DSS-induced colitis (A) Representative flow cytometry dot plots showing the frequencies of colonic lamina propria CD4^+^ T cell subsets across all experimental groups, including CD4^+^ IFN-γ^+^ Th1 cells, CD4^+^ IL-17A^+^ Th17 cells, and CD4^+^ FOXP3^+^ regulatory T (Treg) cells. Numbers within the gated regions indicate the percentage of each subset among total CD4^+^ T lymphocytes. (B–D) Quantitative summary of the frequencies of Th1, Th17, and Treg cells. KA-5817 significantly reduced pathogenic Th1 and Th17 cell frequencies, whereas no statistically significant differences in Treg proportions were detected among groups. (E and F) Concentrations of pro-inflammatory cytokines TNF-α and IL-6 measured in colon tissue homogenates. (G) Relative phosphorylation level of STAT3 (pSTAT3) in colon tissues assessed via colorimetric assay and presented as OD_450_ absorbance values. Data are expressed as mean ± SEM and analyzed using one-way ANOVA. ∗*p* ≤ 0.05, ∗∗*p* ≤ 0.01. ns, no significance.

## Discussion

IBD is a chronic inflammatory disorder driven by dysregulated immune pathways that promote pathogenic Th1 and Th17 polarization, intestinal tissue damage, and fibrosis.^26^ Single-target biologic therapies have improved patient outcomes but remain limited by incomplete efficacy and treatment resistance.^27^^,^^28^ The TL1A-DR3 and IL-12/23 STAT axes are well-validated, non-redundant drivers of intestinal inflammation that act synergistically to amplify pathogenic immune responses.^8^ In this study, we developed and characterized a CLC-based BsAb KA-4316 and its half-life-extended variant KA-5817 that co-target TL1A and IL-12/23p40. Our data demonstrate that dual blockade of these two pathways yields enhanced anti-inflammatory activity *in vitro* and pronounced therapeutic efficacy in humanized mouse colitis models relative to treatment with either single monospecific antibody, supporting this strategy as a promising next-generation approach for IBD. KA-4316 exhibited high binding affinity for both human TL1A and IL-12/23p40 and potently neutralized downstream signaling *in vitro*. Notably, KA-4316 showed selective inhibition of TL1A binding to DR3 relative to DcR3, which may preserve physiological immune regulation while blocking pathological inflammation. In human PBMCs, KA-4316 suppressed IFN-γ production to a greater extent than each parental antibody alone, with efficacy comparable to the combination of the two monospecific antibodies, verifying target-level synergy from dual-pathway inhibition. To enhance clinical translatability, we engineered KA-5817 with the YTE Fc mutation to extend serum half-life. Pharmacokinetic studies in FcRn-humanized mice showed that KA-5817 had a markedly prolonged half-life compared with KA-4316. Evaluation in cynomolgus monkeys further supported favorable pharmacokinetic and safety profiles. These properties are highly desirable for clinical development, as prolonged half-life may enable less frequent dosing and improved patient compliance. The therapeutic efficacy of KA-5817 was validated in a TL1A/IL23A/IL12B triple-humanized mouse model of DSS-induced colitis, which recapitulates human cytokine biology and offers improved clinical relevance compared with conventional mouse models. KA-5817 significantly reduced weight loss, DAI scores, colon shortening, and intestinal edema. Histopathological analysis confirmed preserved mucosal architecture and reduced inflammatory infiltration. These results provide robust proof of concept that dual targeting of TL1A and IL-12/23p40 can effectively ameliorate intestinal inflammation. Compared with monotherapies, the bispecific approach offers several key advantages. While widely adopted for testing mucosal anti-inflammatory therapies, the DSS-induced colitis model is driven primarily by acute epithelial injury and innate immune activation, and cannot fully recapitulate the T cell-mediated Th1/Th17 pathways characteristic of human IBD. The adoptive T cell transfer chronic colitis model exhibits greater pathological relevance. Adoptive transfer of T cells into C57BL/10 RAG2-deficient mice reliably generates persistent inflammation dominated by Th1 and Th17 responses.^29^ Future work will evaluate KA-5817 in this humanized model to verify its translational potential for IBD therapy. Simultaneous blockade of TL1A and IL-12/23 breaks synergistic inflammatory signaling circuits and limits treatment escape driven by compensatory cytokine pathway crosstalk. The CLC design used in KA-4316 and KA-5817 avoids light chain mispairing, simplifies manufacturing, and improves stability and developability relative to other bispecific formats. Collectively, these features support further translational evaluation of KA-5817 as a scalable candidate for IBD therapy.

PF-06480605, a clinical-stage anti-TL1A monoclonal antibody developed by Pfizer, has shown promising efficacy in IBD patients. However, anti-TL1A monotherapy may not fully suppress the complex cytokine milieu in patients with high IL-12/23 activity, leaving a subset of patients with suboptimal responses. Likewise, IL-12/23 inhibitors (e.g., ustekinumab and risankizumab) are not effective in all patients, and resistance can develop through upregulation of TL1A or other compensatory ligands, a limitation that aligns with the core challenge of monotherapies. In this context, the TL1A×IL-12/23p40 BsAb offers a dual advantage: broader immunosuppressive coverage of two non-redundant pathogenic pathways and a reduced likelihood of immune escape through pathway redundancy. This is particularly relevant for patients with refractory IBD, where dual-pathway activation is often observed, and addresses the key unmet need for more effective therapeutic strategies. Notably, PF-07261271, a TL1A×IL-12/23p40 BsAb developed by Pfizer and Roche, currently in phase IIb clinical trials for the treatment of UC, is the first-in-class agent targeting these two pathways. However, PF-07261271 adopts CrossMab technology, which suffers from structural asymmetry that complicates physicochemical and analytical characterization. Additionally, CrossMab is restricted to Fc-containing architectures and faces stringent intellectual property barriers that limit its broad application. In contrast, KA-4316/KA-5817 adopts a CLC format combined with KiH Fc engineering, an approach specifically chosen to address the technical challenges of BsAb development. This design eliminates light chain mispairing, ensures high purity and yield, simplifies manufacturing, and promotes efficient heterodimerization of the two heavy chains. Importantly, this design not only maintains high binding affinity and functional potency but also reduces the risk of immunogenicity and simplifies analytical characterization, key advantages for clinical translation and large-scale production that directly address the practical challenges of BsAb development.

In summary, this study presents a BsAb targeting TL1A and IL-12/23p40, offering a dual-hit approach for the treatment of IBD. KA-4316 and KA-5817 exhibit high affinity for their targets, cross-species activity, excellent pharmacological properties, and robust efficacy *in vitro* and *in vivo*. Compared with existing monotherapies and other BsAb designs, KA-4316/KA-5817 offers distinct advantages in terms of efficacy, safety, manufacturability, and translational potential. These findings establish a strong preclinical foundation for further development toward clinical translation in autoimmune and inflammatory diseases. TL1A×IL-12/23p40 bispecific antibodies represent an attractive direction worthy of further translational investigation for IBD, leveraging simultaneous dual-pathway blockade of two validated pathogenic inflammatory axes to mitigate mucosal inflammation. Preclinical data demonstrate substantial efficacy, favorable safety and PK profiles, as well as robust target engagement. While challenges remain in development, biomarker identification, and long-term safety, TL1A×IL-12/23p40 BsAbs have the potential to transform IBD management by improving remission rates, reducing complications, and enhancing patient quality of life. Further translational and clinical investigations are warranted to fully characterize the therapeutic potential of this dual-targeting agent class for IBD.

### Limitations of the study

This study evaluated *in vivo* efficacy of the BsAb using an acute DSS-induced colitis mouse model. While this model recapitulates core features of intestinal inflammation, it does not fully mimic the chronic, heterogeneous, and progressive immunopathology of human IBD, limiting direct translational extrapolation of murine therapeutic findings. In addition, the pharmacokinetic analysis in cynomolgus monkeys adopted a small sample size, which limited robust interpretation of the antibody’s pharmacokinetic profiles.

## Resource availability

### Lead contact

Requests for further information and resources should be directed to and will be fulfilled by the lead contact, Yu Changyuan (yucy@buct.edu.cn).

### Materials availability

All unique reagents generated in this study are available from the lead contact upon reasonable request for non-commercial research purposes with a completed Materials Transfer Agreement.

### Data and code availability

• Original data reported in this paper have been deposited at Mendeley at https://data.mendeley.com/datasets/6hssp9rs9b/1 and are publicly available as of the date of publication.
• This paper does not report original custom code.
• Any additional information required to reanalyze the data reported in this paper is available from the lead contact upon request.

## Acknowledgments

This research was funded by the Prevention and Control of Emerging and Major Infectious Diseases-10.13039/501100018537National Science and Technology Major Project (grant no. 2025ZD01904100).

## Author contributions

X.L., J.N., and C.Y. conceived and designed the research study. X.L., H.P., F.H., and Y.G. performed the experiments and acquired experimental data. X.L. analyzed and interpreted the data. X.L. drafted the original manuscript. J.N. and C.Y. reviewed, revised, and edited the manuscript. J.N. and C.Y. supervised the entire project and acquired financial support for the study. All authors read and approved the final manuscript.

## Declaration of interests

The authors declare that they have no known competing financial interests or personal relationships that could have appeared to influence the work reported in this paper.

## Declaration of generative AI and AI-assisted technologies in the manuscript preparation process

No generative-AI or AI-assisted technologies were used in the preparation of this manuscript.

## STAR★Methods

### Key resources table

**Table:** 

REAGENT or RESOURCE	SOURCE	IDENTIFIER
**Antibodies**		

KA-4316	This paper	N/A
KA-5817	This paper	N/A
IgG1 Isotype	This paper	N/A
Anti-TL1A mAb	This paper	N/A
Anti-IL-12/23p40 mAb	This paper	N/A
Anti-Human IgG (Fc specific) -Peroxidase antibody	Sigma-Aldrich	A0170; RRID:AB_257868
Goat anti-human IgG-PE	SouthernBiotech	2010–09; RRID:AB_2795568
HRP Conjugated Anti His-Tag Mouse Monoclonal Antibody	CWBIO	CW0285M
FITC anti-mouse CD4 Antibody	Biolegend	100406; RRID:AB_312691
APC anti-mouse IFN-γ	Biolegend	163514; RRID:AB_3097457
PE/Cyanine7 anti-mouse IL-17A	Biolegend	506922; RRID:AB_2125010
PE-conjugated FOXP3 Monoclonal Antibody	eBioscience	12–5773-82; RRID:AB_465936

**Biological samples**		

Human PBMC	Oribiotech	PB003F-C

**Chemicals, peptides, and recombinant proteins**		

Human TL1A/TNFSF15 Protein	ACRO	TLA-H5243
Human IL-12B & IL-12A Heterodimer Protein	ACRO	IL2-H4210
Human IL-23 alpha & IL-12 beta Heterodimer Protein	ACRO	ILB-H52W5
IL-23 alpha & lL-12 beta Heterodimer	Sino Biological	15340-H02H
Cynomolgus/Rhesus macaque TL1A/TNFSF15 Protein	ACRO	TLA-C5241
Recombinant Cynomolgus Monkey IL-12	R&D Systems	10215-CL
Cynomolgus IL-23 alpha & IL-12 beta Heterodimer Protein	ACRO	ILB-C52W3
Cynomolgus IL-23 Protein	Sino Biological	CT146-C08H
Mouse TL1A/TNFSF15 Protein	ACRO	TLA-M5243
Mouse IL-12B&IL-12A Heterodimer Protein	ACRO	IL2-M52W3
Mouse IL-23 alpha&IL-12 beta Heterodimer Protein	ACRO	ILB-M52W7
Human IL-12B/NKSF2/p40 Protein	ACRO	NK2-H52H7
Biotinylated Human IL-12B/NKSF2/p40 Protein	ACRO	NK2-H82E3
Human IL-12B/NKSF2/p40 Protein	ACRO	NK2-H5258
Human IL-12 R beta 1/CD212 Protein	ACRO	ILB-H5255
Human DR3 Protein	ACRO	DR3-H5253
Human DcR3/TNFRSF6B Protein	ACRO	TNB-H5255
Biotinylated Human TL1A/TNFSF15 Protein	ACRO	TLA-H52Q1
HRP-conjugated Streptavidin	BBI	D111054
cycloheximide	Selleck	S7418
Human IL-18 Protein	ACRO	IL8-H5114
dextran sulfate sodium	MP Biomedicals	160110
PMA	Sigma-Aldrich	P8139
Ionomycin, Calcium Salt	Yeasen	50401ES03
Brefeldin A	MCE	HY-16592
TMB Single-Component Substrate Solution	Solarbio	PR1200

**Critical commercial assays**		

Bright-Lite Luciferase Assay System	Vazyme	DD1204
QUANTI-Blue™-SEAP Detection Reagent	Invivogen	rep-qbs
Caspase-Glo 3/7 assay	Promega	G8092
Human IFN-γ ELISA	R&D Systems	DY285B
Pierce Dilution-Free Rapid Gold BCA Protein Assay Kit	Thermo Scientific	A55861
Uncoated Mouse TNF-α (Tumor Necrosis Factor Alpha) ELISA Kit	Elabscience	GEUN-M0103
Uncoated Mouse IL-6 (Interleukin 6) ELISA Kit	Elabscience	GEUN-M0070
PathScan® Phospho-Stat3 (Tyr705) Sandwich ELISA Kit	Cell Signaling Technology	7300

**Deposited data**		

Mendeley Data	Mendeley Data	https://data.mendeley.com/datasets/6hssp9rs9b/1

**Experimental models: Cell lines**		

293T-TNFSF15	Kyinno	KC-0247
293T-cyno-TNFSF15	Kyinno	KC-3544
293T-mouse-TNFSF15	Kyinno	KC-4005
TF1-NFκB-Luciferase-DR3	Kyinno	KC-4317
HEK-Blue™ IL-12 reporter	InvivoGen	hkb-il12
293T-STAT3-Luciferase-IL12Rβ1-IL23R	Kyinno	KC-4519
TF-1	ATCC	CRL-2003
NK92MI	ATCC	CRL-2408

**Experimental models: Organisms/strains**		

B-hFcRn mice	Biocytogen	110001
B-hTL1A/hIL23A/hIL12B mice	Biocytogen	113038
cynomolgus monkeys	Guangxi Bomai Biotechnology	2024121/2024138

**Software and algorithms**		

GraphPad Prism 9.5.1	GraphPad Software	https://www.graphpad.com/scientific-software/prism/
Phoenix 64	Phoenix WinNonlin® Software	https://www.certara.com/software/phoenix-winnonlin/

**Other**		

Octet®AHC2 biosensor chips	Sartorius	18–5142
MycoAlert® Mycoplasma Detection Kit	Lonza	LT07-318

### Experimental model and study participant details

#### Mouse model

For the DSS-induced mouse colitis model, male homozygous TL1A/IL23A/IL12B humanized C57BL/6 mice aged 7–9 weeks were obtained from Biocytogen. Mice were housed in an SPF-grade facility under a 12-h light-dark cycle, with *ad libitum* access to standard chow and drinking water. Environmental conditions were strictly controlled: temperature was maintained at 20°C–26 °C and relative humidity at 30–70%. All animal experimental procedures were approved by the Institutional Animal Care and Use Committee of Pharmalegacy (Animal-use protocol number: IACUC2500388).

For the mouse PK study, female B-hFcRn mice aged 8–12 weeks were purchased from Biocytogen. Mice were housed in an SPF-grade facility under a 12-h light-dark cycle, with *ad libitum* access to standard chow and drinking water. Environmental conditions were strictly controlled: temperature was maintained at 20°C–26 °C and relative humidity at 30–70%. All animal experimental procedures were approved by the Institutional Animal Care and Use Committee of Kyinno (Animal-use protocol number: KY-AUP-N25007).

#### Non-human primate PK study

For the PK study in cynomolgus monkeys, female cynomolgus monkeys aged 3.8–4 years and weighing 2.86–3.39 kg were obtained from Guangxi Bomai Biotechnology. The animals were housed individually in stainless-steel cages within a standard primate housing room. Monkeys were fed twice daily, in the morning and afternoon, with diets supplemented by fruits, vegetables and additional food items. Multiple forms of environmental enrichment, including toys, perches and treats, were provided. Environmental parameters were maintained as follows: temperature of 19.4°C–23.4°C, relative humidity of 35.7–94.0%, and a 12-h light-dark cycle. All animal experimental procedures were approved by the Institutional Animal Care and Use Committee of Anling (Animal-use protocol number: ALSZ-2025–03-44).

#### Cell lines

All cells were cultured at 37 °C in a humidified incubator containing 5% CO_2_. 293T-TNFSF15, 293T-cyno-TNFSF15 and 293T-mouse-TNFSF15 cells were maintained in DMEM supplemented with 10% fetal bovine serum (FBS), with the addition of 0.5 μg/mL puromycin for 293T-TNFSF15 cells and 1 μg/mL puromycin for the other two cell lines. TF1-NFκB-Luciferase-DR3 cells were cultured in RPMI-1640 medium supplemented with 10% FBS, 5 ng/mL GM-CSF, 100 μg/mL Hygromycin B and 1 μg/mL puromycin. The HEK-Blue IL-12 reporter cell line was grown in DMEM containing 10% FBS, 100 μg/mL Normocin and HEK-Blue Selection reagent. 293T-STAT3-Luciferase-IL12Rβ1-IL23R cells were maintained in DMEM supplemented with 10% FBS, 1 μg/mL puromycin and 140 μg/mL Hygromycin B. TF-1 cells were cultured in RPMI-1640 medium supplemented with 10% FBS and 2 ng/mL GM-CSF. NK92MI cells were cultured in StemCell H5100 complete medium. Isolated human PBMCs were maintained in RPMI-1640 medium supplemented with 10% FBS.

All human cell lines were authenticated via short tandem repeat (STR) profiling and tested negative for mycoplasma contamination using the MycoAlert Mycoplasma Detection Kit. Healthy human PBMCs (donor ID: T0187, 21-year-old Asian male, blood type O+) were screened negative for HIV, HBV, HCV and syphilis. Bacterial and mycoplasma testing of the PBMC preparation were negative.

### Method details

#### Antibody design and engineering

TL1A×IL-12/23p40 BsAb KA-4316 were constructed using the KiH heterodimerization strategy. VH and VL sequences specific to human TL1A and IL-12/23p40 were isolated via hybridoma screening. A common light chain was used to simplify bispecific assembly. KA-5817 was generated by introducing Fc mutations (M252Y/S254T/T256E) into KA-4316 to enhance FcRn binding and prolong serum half-life.

#### ELISA binding assays

Antigen binding ELISA was performed by coating 96-well plates overnight at 4°C with 50 ng recombinant human, cynomolgus monkey and mouse cytokines (IL-12, IL-23 and TL1A). After blocking with 2% BSA in PBS, serial dilutions of BsAb KA-4316 were added and incubated at RT for 1 h. Detection was done using Anti-Human IgG (Fc specific)-Peroxidase antibody produced in goat and TMB substrate. Absorbance at 450 nm was read using a microplate reader (Clario Star Plus, BGM). EC_50_ values were calculated using a 4-parameter logistic regression (GraphPad Prism v9).

#### Sandwich ELISA binding assays

Two reciprocal sandwich ELISA assays were performed to assess the dual-antigen binding capability of the KA-4316. 96-well plates were coated overnight at 4 °C with either recombinant human TL1A-His or human IL-12B/p40-Fc proteins. All wells were blocked with 2% BSA in PBS. Antibody samples were serially diluted. After antibody incubation, biotin-conjugated IL-12B/p40-His was added to TL1A-coated wells and detected with 1:5000 HRP-streptavidin. Conversely, unlabeled TL1A-His was added to IL-12B/p40-Fc-coated wells and detected with 1:5000 HRP-conjugated anti-His-tag antibody. After incubation with TMB substrate, OD450 absorbance values were recorded, and EC_50_ values were calculated using a 4-parameter logistic regression.

#### Flow cytometry cell-surface binding assays

293T cells stably overexpressing human TL1A, Cyno TL1A or mouse TL1A were collected, subjected to blocking treatment, and incubated with serially diluted BsAb KA-4316, 293T parental cell as a negative control. Cells were subsequently stained with a fluorophore-conjugated secondary antibody Goat anti-human IgG-PE and analyzed using a flow cytometer (Attune NxT Acoustic Focusing Cytometer, Invitrogen). MFI values were plotted against antibody concentrations to determine the cellular binding EC_50_ values.

#### TL1A/receptors competition ELISA

KA-4316 was assessed for its ability to inhibit binding of TL1A to its receptors in a competition ELISA. 200 ng Human DR3 Protein or 200 ng Human DcR3/TNFRSF6B Protein were coated onto a 96-well plate at 4°C overnight, and then next day plates were washed and blocked in a solution of PBS with 2% w/v BSA. Serially diluted KA-4316 was pre-incubated with 0.2 μg/mL Biotinylated Human TL1A/TNFSF15 Protein for 30 min then added to the DR3-Fc or DcR3-Fc coated wells. Bound TL1A was detected using HRP-conjugated Streptavidin 1:5000 and visualized with TMB Single-Component Substrate Solution. TMB reaction was stopped with 1 M hydrochloric acid. The data were normalized by expression as a percentage of maximum binding of TL1A to receptor in the absence of KA-4316.

#### IL-12/23p40/IL-12Rβ1 competition ELISA

KA-4316 was assessed for its ability to inhibit binding of IL-12/23p40 to IL-12Rβ1 in a competition ELISA. 200 ng Human IL-12 R beta 1 Protein was coated onto a 96-well plate at 4°C overnight, and then next day plates were washed and blocked in a solution of PBS with 2% w/v BSA. Serially diluted KA-4316 was pre-incubated with 1.2 μg/mL IL-12 protein or 1 μg/mL IL-23 protein for 30 min then added to the IL-12Rβ1 coated wells. Bound IL-12 or IL-23 was detected using HRP Conjugated Anti His-Tag Mouse Monoclonal Antibody 1:2000 and visualized with TMB Single-Component Substrate Solution. TMB reaction was stopped with 1 M hydrochloric acid. The data were normalized by expression as a percentage of maximum binding of IL-12 or IL-23 to receptor in the absence of KA-4316.

#### Biolayer interferometry

A Molecular interaction analyzer (OCTETRED96E, ForteBio) was used for Biolayer Interferometry analysis. Octet®AHC2 biosensor chips which are pre-immobilized with a new anti-human Fc-specific antibody were employed for the immobilization of IgG antibodies. Prior to the experiment, the chips were equilibrated in 0.01M PBS pH 7.4, 0.01% BSA, 0.04% (v/v) tween 20, and 0.5 mg/mL BSA buffer to ensure proper surface hydration and remove any contaminants. Briefly, the sensor tips were incubated in a solution of 5 μg/mL KA-4316 in PBS for 180 s at room temperature. After antibody immobilization, the sensor surface was washed with PBS to remove unbound antibody. Following immobilization, the sensor was placed in the BLI system and the baseline signal was recorded by immersing the sensor tips in PBS for 120 s. This step was essential to ensure that the baseline signal was stable before the introduction of the antigen. To evaluate the binding kinetics, various concentrations of the antigen (ranging from 6.25 nM to 100 nM) were injected over the immobilized antibody surface. TL1A antigen and IL-12/23p40 antigen, respectively. The antigen was prepared in PBS buffer, and the association phase was monitored for 180 s. The sensor was then washed with PBS to allow for dissociation of the bound antigen. After the antigen injection, the dissociation phase was recorded by allowing the antigen to dissociate from the antibody over a period of 300 s in PBS. The dissociation phase was used to determine the off-rate (*k*_d_) of the interaction. The data were analyzed using ForteBio Data Analysis Software. The binding kinetics were fitted to a 1:1 binding model to determine the association rate constant (*k*_on_) and dissociation rate constant (*k*_off_) for each antigen concentration. The equilibrium dissociation constant (*K*_D_) was calculated.

#### TL1A signal transduction inhibition assay

TF1-NFκB-Luciferase-DR3 cells (KC-4317, Kyinno) were stimulated with 100 ng/mL TL1A protein pre-mixed with antibodies for 6 h at 37°C. Luciferase activity was measured using Bright-Lite Luciferase Assay System.

#### IL-12 signal transduction inhibition assay

HEK-Blue IL-12 reporter cells were used. Cells were incubated with 10 ng/mL IL-12 protein pre-mixed with antibodies for 24 h at 37°C. Secreted alkaline phosphatase was detected using QUANTI-Blue-SEAP Detection Reagent and OD630 measurement.

#### IL-23 signal transduction inhibition assay

293T-STAT3-Luciferase-IL12Rβ1-IL23R cells were stimulated with 40 ng/mL IL-23 protein pre-mixed with antibodies for 6 h at 37°C. Luciferase activity was measured using Bright-Lite Luciferase Assay System.

#### Apoptosis inhibition assay

TF-1 cells (Human erythroblast cell line) were treated with 125 ng/mL TL1A protein the presence of 20 μg/mL cycloheximide. Antibodies were pre-incubated with TL1A protein before being added to the cells. After 6 h, Caspase-Glo 3/7 assay was performed to measure apoptotic activity.

#### IFN-γ secretion assays

NK92MI cells (Human natural killer cell line) were stimulated with 0.3 ng/mL IL-12 protein. Antibodies were pre-incubated with IL-12 protein before being added to the cells. After 24 h incubated in 37°C, supernatants were collected, and IFN-γ levels were measured using ELISA (R&D Systems, DY285B).

PBMCs (Human peripheral blood mononuclear cell) were stimulated with 0.5 ng/mL IL-12 protein, 1 ng/mL IL-18 protein, and 20 ng/mL TL1A protein. Antibodies were pre-incubated with three proteins before being added to the PBMCs. After 48 h incubated in 37°C, supernatants were collected, and IFN-γ levels were measured using ELISA.

#### Pharmacokinetic of TL1A and IL-12/23p40 bispecific antibodies in hFcRn mice

Female B-hFcRn mice aged 8–12 weeks were purchased from Biocytogen. All mice were randomly divided into two groups with 3 mice in each group. Mice in Group 1 and Group 2 were intravenously administered with KA-5817 and KA-4316 at a single dose of 10 mg/kg (200 μg per mouse, 200 μL per mouse), respectively. Blood samples were collected from the mice at 0 min, 15 min, 1 h, 4 h, 1 day (d), 3 days, 5 days, 7 days, 10 days, 14 days, 21 days and 28 days post-administration. Plasma was separated from the blood samples for subsequent PK detection, and all plasma samples were diluted 5000-fold before detection. PK parameters were calculated based on the plasma antibody concentration detected by ELISA and were calculated by non-compartmental analysis (NCA) using Phoenix 64 software.

#### Pharmacokinetic of KA-5817 in cynomolgus monkeys

Two female cynomolgus monkeys (*Macaca fascicularis*), aged 3.8–4 years and weighing 2.86–3.39 kg were obtained from Guangxi Bomai Biotechnology with animal qualification numbers No.2024121 and No.2024138. The animals were housed individually in stainless steel cages in a standard monkey rearing room with controlled environmental conditions: temperature 19.4°C–23.4°C, relative humidity 35.7–94.0%, and a 12 h light/dark cycle (except for pharmacokinetic blood collection days). The animal welfare protocol was approved by the Institutional Animal Care and Use Committee (IACUC) and followed national ethical guidelines. The monkeys were divided into two groups (*n* = 1 animal per group) and received KA-5817 at doses of 3 mg/kg (Group 1) and 10 mg/kg (Group 2), respectively. The dosing volume was 2 mL/kg for both groups, administered by a single intravenous bolus injection. Blood samples for PK analysis were collected from the anterior limb vein at the following time points: pre-dosing (0 h) on D1; 5 min, 1 h, 2 h, 8 h, 24 h, 72 h, 120 h, 168 h, 336 h, 504 h post-dosing on D1. Approximately 1 mL of blood was collected into EDTA-K2 anticoagulant tubes, gently mixed, and placed on ice. Plasma was separated by centrifugation (4°C, 2000×g, 10 min) within 1 h of collection, and at least 400 μL of plasma was obtained. Plasma samples were analyzed of KA-5817 concentration using ELISA method. Pharmacokinetic parameters were calculated by NCA using Phoenix 64 software.

#### DSS-induced mouse colitis model

7–9 weeks old homozygous TL1A/IL23A/IL12B humanized C57BL/6 mice were housed in an SPF facility with a 12-h light/dark cycle and *ad libitum* access to standard chow and water. All experimental procedures were approved by the IACUC and performed in accordance with institutional guidelines. Acute colitis was induced by administering 2% (w/v) DSS (molecular weight 36–50 kDa) dissolved in autoclaved drinking water *ad libitum* from day 0 (D0) to day 6 (D6). Body weight, stool consistency, and rectal bleeding were monitored daily to assess disease activity. Mice were randomly assigned to six groups (*n* = 6 animals per group). Antibodies were administered via tail vein intravenous injection on Days 0, 3 and 7 at a consistent total dose of 20 mg/kg: IgG1 isotype (20 mg/kg), anti-TL1A mAb (20 mg/kg), anti-IL-12/23p40 mAb (20 mg/kg), Combo (10 mg/kg anti-TL1A plus 10 mg/kg anti-IL-12/23p40 mAb), and KA-5817 (20 mg/kg). The naive group remained untreated without DSS exposure or antibody intervention. The antibodies were diluted in sterile PBS to a final volume of 100 μL per injection. On day 9 (D9), all mice were euthanized by cervical dislocation under isoflurane anesthesia. Colonic tissues were harvested, rinsed with cold PBS, and fixed in 4% paraformaldehyde (PFA) for histological processing.

#### Isolation of colonic lamina propria immune cells

Colonic tissues were dissected, rinsed with ice-cold PBS to clear fecal residues, and freed of adipose tissues. The cleaned colons were longitudinally incised and minced into small fragments. To remove epithelial cells, tissue fragments were incubated in pre-cooled PBS containing 5 mM EDTA and 2% FBS at 37 °C for 20 min with gentle shaking. After thorough washing to eliminate epithelial components, tissues were digested in RPMI 1640 medium supplemented with 2% FBS, 1.5 mg/mL collagenase IV and 0.5 mg/mL DNase I at 37 °C for 45 min under continuous shaking. The digested suspension was filtered through a 70 μm cell strainer to discard undigested debris. Immune cells were isolated via a discontinuous Percoll density gradient and harvested for subsequent flow cytometric staining.

For detection of IFN-γ-producing Th1 and IL-17A-producing Th17 cells, isolated Immune cells were resuspended in complete RPMI 1640 medium containing 10% FBS and stimulated with 50 ng/mL PMA, 1 μg/mL ionomycin and 5 μg/mL brefeldin A for 3 h at 37 °C in 5% CO. After stimulation, cells were harvested and stained with surface antibodies including FITC anti-mouse CD4 Antibody for 15 min at room temperature. Subsequently, cells were fixed and permeabilized. Intracellular IFN-γ and IL-17A were stained with corresponding fluorophore-conjugated antibodies (APC anti-mouse IFN-γ and PE/Cyanine7 anti-mouse IL-17A) at room temperature for 30 min in the dark. After washing, cells were resuspended and analyzed by flow cytometry. For regulatory T cell detection, freshly isolated immune cells were stained CD4 and CD25. After surface staining, cells were fixed and permeabilized. Intracellular Foxp3 staining was performed by PE-conjugated FOXP3 Monoclonal Antibody.

For tissue cytokine and pSTAT3 measurement, colon tissue segments were collected and rinsed with cold PBS to remove fecal residues. Tissues were weighed and homogenized in ice-cold lysis buffer supplemented with protease and phosphatase inhibitor cocktails. After thorough homogenization, samples were centrifuged at 12,000 × g for 15 min at 4 °C. The supernatants were harvested, and total protein concentrations were quantified using a BCA protein assay kit. The levels of pro-inflammatory cytokines TNF-α, IL-6 and phosphorylation level of STAT3 (pSTAT3) in colon tissue homogenates were measured by ELISA kits (Uncoated Mouse TNF-α (Tumor Necrosis Factor Alpha) ELISA Kit; Uncoated Mouse IL-6 (Interleukin 6) ELISA Kit; PathScan® Phospho-Stat3 (Tyr705) Sandwich ELISA Kit).

### Quantification and statistical analysis

#### Statistical analysis in PK parameters calculation

PK parameters including maximum observed concentration (Cmax), terminal elimination half-life (*t*_1/2_) and area under the concentration-time curve from time zero to infinity (AUC_0-∞_) were calculated by non-compartmental analysis using Phoenix 64 software.

#### Statistical analysis in animal study

Statistical analyses were conducted using GraphPad Prism 9.5.1. Longitudinal time-course data were analyzed by two-way repeated-measures ANOVA, followed by Tukey’s multiple comparisons test to evaluate intergroup differences at individual time points. Comparisons across multiple groups at a single endpoint were conducted via one-way ANOVA with Tukey’s post-hoc multiple-comparisons test. All computed statistical outputs, including F-statistics, degrees of freedom, R-squared values, exact Tukey-adjusted *p*-values for all pairwise comparisons, and the number of biological units per group are compiled and available in Data S1 (Compiled statistical analysis outputs for Figures 6B–6E and 7B–7G). Data are presented as the mean ± standard error of the mean (SEM). Statistical significance was defined as ∗*p* ≤ 0.05, ∗∗*p* ≤ 0.01, ∗∗∗*p* ≤ 0.001. ns, no significance.

## Supplemental information

Document S1. Figures S1–S3

Data S1. Compiled statistical analysis outputs for Figures 6B–6E and 7B–7G
