# Supplementary material for: Bispecific antibodies targeting TL1A and IL-12/23p40 suppress pathogenic Th1/Th17 responses and ameliorate colitis in mice
Source: iScience. 2026 Oct 9;29(11):117795. doi: 10.1016/j.isci.2026.117795 (PMC13677191; doi:10.1016/j.isci.2026.117795)
Supplement: Supplementary file 1 [file mmc1.pdf]

iScience, Volume *29*

Supplemental information

Bispecific antibodies targeting TL1A and IL-12/23p40 suppress pathogenic Th1/Th17 responses and ameliorate colitis in mice

Xia Li, Hao Peng, Feng Hao, Yi Gu, Jinying Ning, and Changyuan Yu

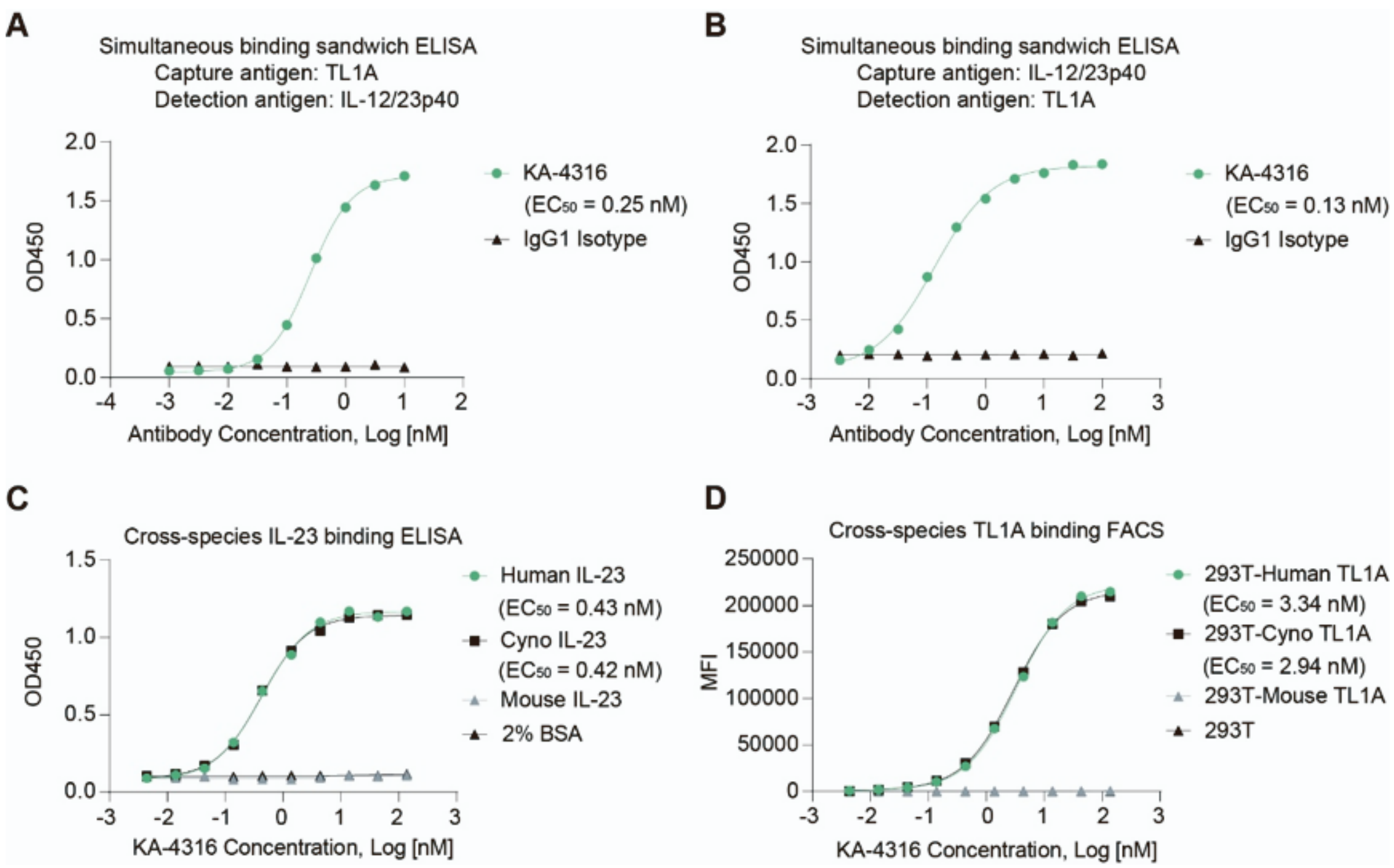

Figure S1. Binding profiles of bispecific antibody KA-4316

(A-B) Simultaneous dual-antigen binding assessed by sandwich ELISA. (A) TL1A was immobilized as capture antigen, and soluble IL-12/23p40 was used for detection. (B) IL-12/23p40 was immobilized as capture antigen, and soluble TL1A was used for detection. KA-4316 exhibited concentration-dependent simultaneous binding to both antigens, while IgG1 isotype control showed negligible signal. Corresponding EC_{50} values are indicated.

(C) Cross-species binding to IL-23 measured by ELISA. KA-4316 binds human and cynomolgus IL-23 with comparable potency, but displays minimal binding to mouse IL-23. EC_{50} values for human and cynomolgus IL-23 are provided. 2% BSA served as blank control.

(D) Cross-species binding to cell-surface TL1A determined by flow cytometry (FACS). 293T cells overexpressing human or cynomolgus TL1A were incubated with serially diluted KA-4316. Concentration-dependent binding to human and cynomolgus TL1A was observed with similar EC_{50} values. No obvious binding to mouse TL1A or empty 293T cell control was detected. MFI, mean fluorescence intensity.

Dose-response curves were fitted by nonlinear regression using a four-parameter logistic model. EC_{50} values are reported with curve-fitted 95% confidence intervals.

A

Fraction affected (Fa)	Monospecific antibody combo CI value	Interaction classification
0.5 (EC50)	0.072	Synergy
0.75 (EC75)	0.095	Synergy
0.9 (EC90)	0.133	Synergy

B

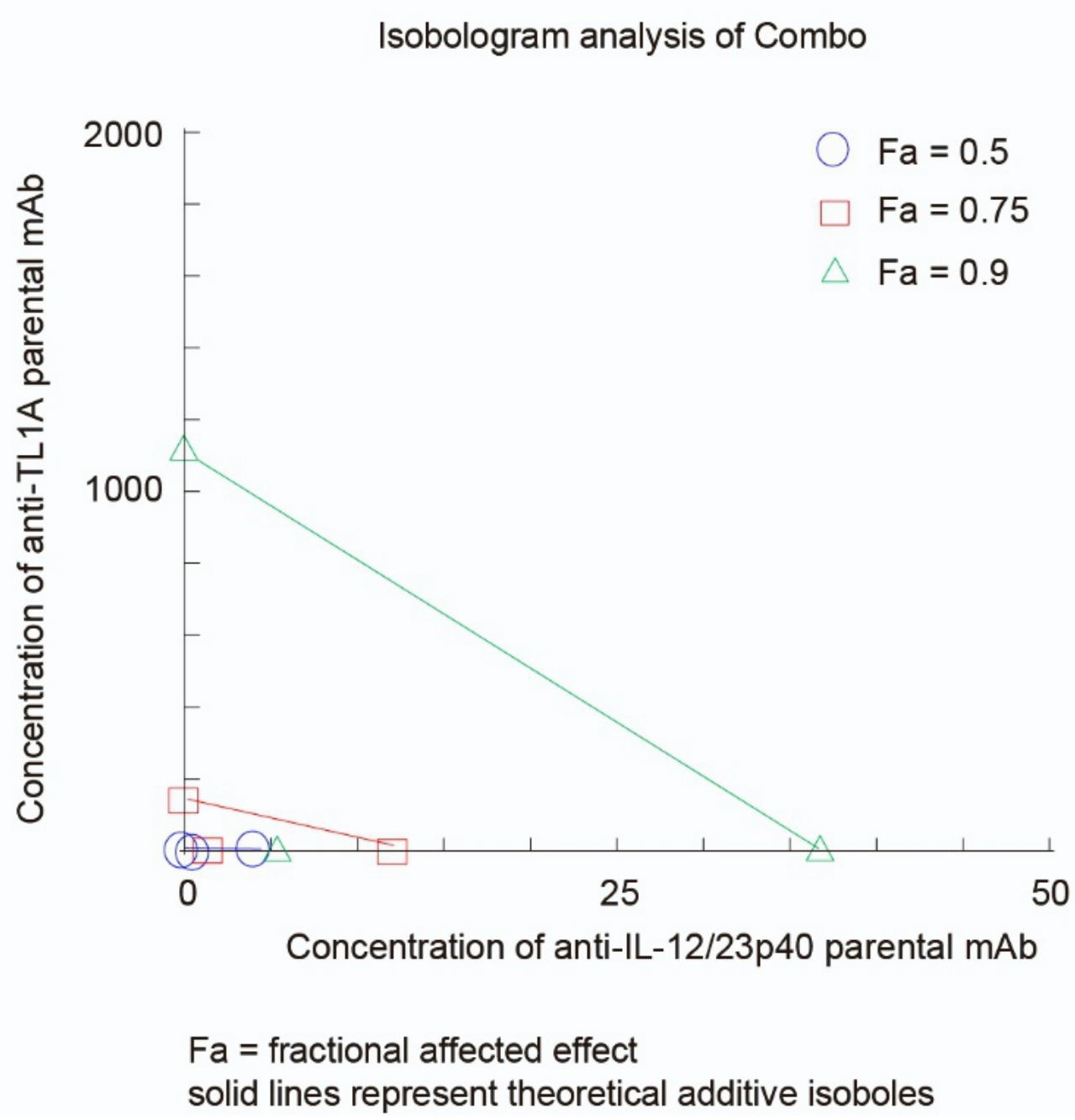

Figure S2. Quantitative synergy analysis of the combination of two monospecific antibodies

(A) Combination Index (CI) values calculated by the Chou-Talalay method at fractional effect (Fa) levels of 0.5, 0.75 and 0.9. CI values for the monospecific antibody combination were consistently below 1, indicating strong synergistic pharmacological activity. CI < 1, synergy; CI = 1, additivity; CI > 1, antagonism.

(B) Isobologram analysis of the monospecific antibodies combination at Fa = 0.5, 0.75 and 0.9. All experimental combination data points fall below the theoretical additive isobole lines, providing independent graphical confirmation of synergistic interaction.

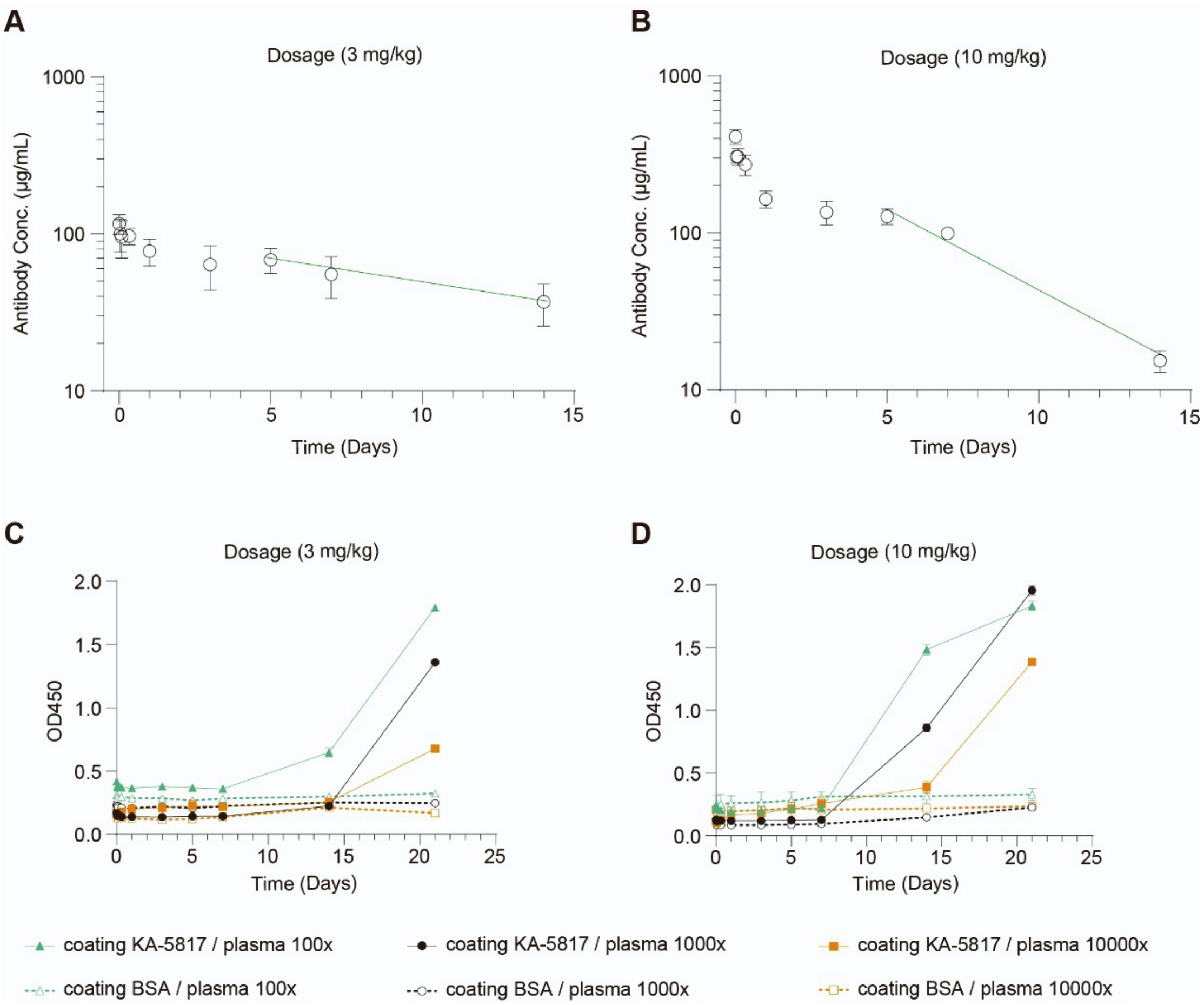

Figure S3. Time-course serum concentration profiles and ADA signals in cynomolgus monkeys after KA-5817 dosing

(A-B) Concentration-time curves after single intravenous injection at 3 mg/kg and 10 mg/kg. Terminal elimination phase fitting for $T_{1/2}$ utilized sampling data obtained on Day 5, 7 and 14; Day 21 data were omitted to avoid distortion caused by ADA interference. Error bars represent 95% confidence intervals. Plotted values are ELISA technical replicates reflecting only assay technical variation and not inter-animal variability. Data are presented with 95% confidence intervals of the mean. No statistical analysis was performed.

(C-D) 3 mg/kg and 10 mg/kg single-dose groups monitored over 21 days. Due to the lack of KA-5817-specific ADA reference standard for absolute quantification, relative ADA levels were determined by OD450 absorbance of serially diluted plasma on KA-5817-coated plates; BSA-coated wells serve as blank controls. Higher and earlier ADA signals are observed in the 10 mg/kg cohort, correlating with reduced drug half-life at high dose. Data are mean ± SEM.
